# Exploration of the intracellular chiral metabolome in pediatric BCP-ALL: a pilot study investigating the metabolic phenotype of IgH locus aberrations

**DOI:** 10.3389/fonc.2024.1413264

**Published:** 2024-08-05

**Authors:** Meghan Collins, Ruggiero Gorgoglione, Valeria Impedovo, Xingxin Pan, Sathyaseelan Chakkarai, S. Stephen Yi, Alessia Lodi, Stefano Tiziani

**Affiliations:** ^1^ Department of Nutritional Sciences, College of Natural Sciences, The University of Texas at Austin, Austin, TX, United States; ^2^ Dell Pediatric Research Institute, Dell Medical School, The University of Texas at Austin, Austin, TX, United States; ^3^ Department of Oncology, Livestrong Cancer Institutes, Dell Medical School, The University of Texas at Austin, Austin, TX, United States; ^4^ Interdisciplinary Life Sciences Graduate Programs, College of Natural Sciences, The University of Texas at Austin, Austin, TX, United States; ^5^ Oden Institute for Computational Engineering and Sciences, and Department of Biomedical Engineering, The University of Texas at Austin, Austin, TX, United States; ^6^ Department of Pediatrics, Dell Medical School, The University of Texas at Austin, Austin, TX, United States

**Keywords:** pediatric leukemia, immunoglobulin heavy chain locus, liquid chromatography high resolution tandem mass spectrometry, untargeted chiral metabolomics, transcriptomics

## Abstract

**Background and aims:**

Aberrations in the immunoglobulin heavy chain (IgH) locus are associated with poor prognosis in pediatric precursor B-cell acute lymphoblastic leukemia (BCP-ALL) patients. The primary objective of this pilot study is to enhance our understanding of the IgH phenotype by exploring the intracellular chiral metabolome.

**Materials and methods:**

Leukemia cells were isolated from the bone marrow of BCP-ALL pediatric patients at diagnosis. The samples’ metabolome and transcriptome were characterized using untargeted chiral metabolomic and next-generation sequencing transcriptomic analyses.

**Results:**

For the first time D- amino acids were identified in the leukemic cells’ intracellular metabolome from the bone marrow niche. Chiral metabolic signatures at diagnosis was indicative of a resistant phenotype. Through integrated network analysis and Pearson correlation, confirmation was obtained regarding the association of the IgH phenotype with several genes linked to poor prognosis.

**Conclusion:**

The findings of this study have contributed to the understanding that the chiral metabolome plays a role in the poor prognosis observed in an exceptionally rare patient cohort. The findings include elevated D-amino acid incorporation in the IgH group, the emergence of several unknown, potentially enantiomeric, metabolites, and insights into metabolic pathways that all warrant further exploration.

## 1 Introduction

According to the American Cancer Society, following accidents, cancer is the second leading cause of death in children 1 to 14 years of age (1, 2). Acute Lymphoblastic Leukemia (ALL) represents more than 30% of all pediatric cancers with B-cell precursor acute lymphoblastic leukemia (BCP-ALL) being the most common subtype. Diagnosis and prognosis involve a comprehensive assessment of clinical symptoms, blood tests, and, importantly, cytogenetic analysis (3–9). Identifying the chromosomal aberrations within the leukemia cells is essential for tailoring effective treatment plans, predicting prognosis, and advancing research efforts to improve outcomes for affected children (3–5, 7–10). Fluorescent *in situ* hybridization (FISH) is routinely used to detect aberrations such as translocations, deletions, fusions, and trisomies from cells isolated from bone marrow aspirates at diagnosis (9, 11, 12). Many recurrent cytogenetic aberrations in BCP-ALL have been shown to correlate with patient outcomes and are recognized by the World Health Organization (4–10, 12–17). This includes translocations, excluding physiological V-D-J IgH rearrangements, involving the IgH locus that have been consistently associated with unfavorable outcomes in patients (7, 12, 18). While specific translocations and other aberrations at the IgH locus have been observed in BCP-ALL patients, their clinical implications remain an ongoing area of research in BCP-ALL as well as other hematological malignancies (19–27).

While cytogenetic information can provide detailed insights into the genetic alterations, aiding in risk stratification, it cannot offer a functional perspective on how chromosomal aberrations may impact cellular metabolism and successful response to standard-of-care treatment (28). Currently, BCP-ALL patients with and without an IgH aberration are treated according to the same protocol that includes PEG-L-asparaginase (PEG-L-ASP) along with other chemotherapy agents (14, 18, 29, 30). This treatment strategy specifically targets extracellular L-asparagine and glutamine as the lack of asparagine synthase (ASNS) in B-cell leukemia cells results in an increased dependency on extracellular L-asparagine for cell survival and proliferation (14, 28–31). Taking advantage of this unique metabolic vulnerability results in diminishing leukemia cell’s ability to synthesize key proteins, therefore compromising the ability to proliferate (28–31). This represents one of several instances where a cancer treatment targets a pathway reliant on chiral metabolites. Given the evolving comprehension that D-amino acids serve as biomarkers with diagnostic significance for various diseases including cancer, it is imperative to be able to simultaneously assess both chiral and achiral metabolites in a single sample (32). Moreover, given the poor prognosis in patients with IgH translocations, an increased understanding of the metabolic consequences associated with carrying any IgH-aberration is needed to evaluate alternative therapeutic approaches.

Metabolomics involves measuring low-molecular-weight molecules within cells and biological systems. It is increasingly employed to profile phenotypic and molecular downstream effects of gene and drug perturbations, both *in vitro* and *in vivo*, as well as in clinical samples (33–36). However, traditionally metabolomics data have been acquired without discriminating the metabolic stereospecificity. We recently developed a liquid chromatography-high resolution tandem mass spectrometry (LC-HR-MS/MS)-based untargeted chiral metabolomics (UCM) methodology that allows to discriminate metabolic stereospecificity with high sensitivity and selectivity (37). This novel strategy utilizes diacetyl-tartaric anhydride (DATAN) for the simultaneous conversion of, for example, the two enantiomers of amino acids (AA) or hydroxy acids (HA) into four diastereomers (37, 38). Based on the order of elution, the D- and L- enantiomers of a metabolite can be discriminated and relatively quantified (37). This approach was used to investigate the enantioselective metabolic profile of the bone marrow microenvironment and the peripheral blood plasma of pediatric acute myeloid leukemia (AML) patients and revealed significant alterations in response to chemotherapy (37). The developed method has also been applied to investigate circulating chiral metabolic biomarkers associated with tumor hypoxia and early response to treatment in patients with glioblastoma (39). The ability to discriminate the enantiomeric forms of 2-hydroxyglutarate (2-HG) have led to the successful development of targeted therapeutic treatments, demonstrating the importance of the ability to differentiate enantiomers of a metabolite (37, 40–43).

This pilot study aims to demonstrate that a shared chiral “metabolic phenotype” exists among pediatric BCP-ALL patients with IgH-aberrations, independent of the specific “gene subtypes” (44). The aberrations carried by these patients include a translocation at 14q32, trisomy, IgH::EPOR, and deletions. Additionally, it endeavors to investigate previously overlooked factors within the chiral metabolome that may contribute to treatment challenges faced by pediatric patients. Employing the advanced methodology of UCM has revealed for the first time, to the best of our knowledge, the intracellular presence of D-amino acids and other chiral metabolites within the bone marrow. Our approach, encompassing both molecular and genetic aspects, holds promise in contributing to the advancement of therapeutic strategies as we strive toward improved patient outcomes and survival rates in this population.

## 2 Materials and methods

### 2.1 Reagents and chemicals

LC-MS grade methanol (MeOH), acetonitrile (ACN), ammonium formate, chloroform, formic acid (FA), glacial acetic acid, and (+)-diacetyl-L-tartaric anhydride (with a purity exceeding 98%, Fisher Scientific), along with Pierce FlexMix Calibration Solution were procured from Fisher Scientific (Pittsburgh, PA, USA) (-).-diacetyl-D-tartaric anhydride (with a purity exceeding 97%) was purchased from TCI Chemicals (Chuo-ku, Tokyo, Japan). Additional supplies, including Hanks’ Balanced Salt Solution (HBSS) and Ficoll-Paque Plus, were also sourced from Thermo Fisher Scientific. Isotopically labeled compounds such as D4-alanine, D3-serine, D2-glycine, D3-aspartic acid, D4-L-lysine, D3-DL-glutamic acid, D5-glutamine, and D5-L-tryptophan were procured from Cambridge Isotope Laboratories (Tewksbury, MA, USA).

### 2.2 Declaration of ethical approval

Clinical inquiries were carried out adhering to the principles outlined in the Declaration of Helsinki. Approval for human studies was granted by the Institutional Review Boards (IRB) of both UT Austin Health Science (2013–06-0047, 2018–05-0129) and Ascension Seton (CR-13–161). Prior to specimen collection and study inclusion, written informed consent and parental/guardian permission were obtained.

### 2.3 Patients and sample collection

The clinical team at the Children’s Blood and Cancer Center (CBCC) at the Dell Children’s Medical Center in Austin, Texas, recruited patients and collected the primary samples used in this research study. Written and informed consent, as well as parental/guardian permission, were obtained for the collection of bone marrow (BM) aspirates at the time of diagnosis, following Institutional Review Board (IRB) guidelines. Eligibility for the study required patients to be between one and 21 years old at the time of diagnosis.

Clinical information, including age, gender, weight, body mass index (BMI), race, ethnicity, risk classification, and histopathology (assessed through flow cytometry, karyotype, and fluorescence *in situ* hybridization (FISH) where applicable), was reported post-diagnosis. Treatment for all patients adhered to the protocols established by the Children’s Oncology Group (COG), determined by the specific diagnosis and risk assessment, as outlined in **Supplementary Table S1**. Patients diagnosed with precursor B-cell acute lymphoblastic leukemia (BCP-ALL) followed COG protocol AALL0932 (standard risk) or AALL1131 (high risk). Generally, induction therapy consisted of a combination of cytarabine, vincristine, methotrexate, PEG-L-ASP, and a glucocorticoid (either dexamethasone or prednisone). High risk patients also received daunorubicin. Clinical risk assessment at diagnosis considered factors such as age, sex, white blood cell count, and the presence of leukemia cells in cerebrospinal fluid. A limited number of cytogenetic markers were also utilized when available. A Wilcoxon rank sum test was conducted for the characteristics of age, weight, and BMI. Any p-value greater than 0.05 was deemed not significant between the groups.

Heparinized vacuum blood collection tubes were utilized for the collection of patient specimens (Becton, Dickinson and Company, Franklin Lakes, New Jersey, USA). Leukemia cells were isolated from bone marrow (BM) using a modified Ficoll-Paque protocol as previously described (45, 46). In summary, the BM aspirate was mixed in a 1:1 ratio with ice-cold Hanks’ Balanced Salt Solution (HBSS) and carefully layered over 3 mL of Ficoll-Paque Plus solution. Subsequently, the specimens underwent centrifugation at 400 g without a break for 30 minutes at 18°C. The collected mononuclear cell layer was then washed twice with 10 mL of ice-cold HBSS. Following this, the cells were counted, pelleted, rapidly frozen in liquid nitrogen, and stored at -80°C for subsequent analysis.

### 2.4 Metabolomics sample preparation

Metabolites were extracted from cell pellets by modified Bligh-Dyer as we previously reported (47, 48). In brief, primary cell pellets were extracted with ice cold 1:1 water:methanol and equal parts chloroform. Butylated hydroxytoluene was added to preserve metabolites susceptible to oxidation. Polar fractions were isolated and spiked with a mix of internal standards solution to achieve a final concentration of 1.5 ppm following derivatization. Samples were then evaporated to dryness at 4°C in a CentriVap refrigerated vacuum concentrator (Labconco, Kansas City, MO, USA) and kept at -80°C until analysis. Derivatization was completed as previously described by the addition of 50 μL of a solution containing (+)-DATAN (75 mg/mL) in acetonitrile: glacial acetic acid (ACN: AcOH, volume/volume 4:1) to the sample [26]. The mixture was vigorously vortexed and then placed in a heating block at 75°C for 2 hours. After derivatization, the reaction mixture was dried in a CentriVap refrigerated vacuum concentrator at 4°C, reconstituted in 100 μL ACN: AcOH (4:1) and transferred into Eppendorf tube and centrifuged at 4°C and 10,000 rpm for 10 minutes. Pulling from the supernatant, 75 μL was transferred to a Certified QSertVial LC-MS vial from Sigma-Aldrich. To ensure analytical quality, control samples (QC) representing an equivalent concentration to the study samples were prepared before derivatizing plasma samples. The QCs included IgH-aberration-positive and -negative at diagnosis as well as an all-encompassing pooled QC. For the group QCs, a 10 μL aliquot from each sample was combined while for the pooled QC 5 μL aliquot from each sample was combined. The pooled QC was then split into two equal portions, one portion was labeled with (+)-DATAN, while the other was labeled with (−)-DATAN, following the derivatization procedure, and subsequently subjected to LC-MS analysis.

### 2.5 Liquid chromatography-mass spectrometry untargeted chiral metabolomic analysis

Chiral metabolic analysis was conducted using a Orbitrap IQ-X Tribrid mass spectrometer (Thermo Scientific, Waltham, MA) coupled to a Vanquish Flex ultrahigh-pressure liquid chromatography (UHPLC) system as previously described (37). Chromatographic separation was accomplished on an ACQUITY BEH C18 150 × 2.1 mm (1.7 μm, 130 Å) column from Waters (Milford, MA). A gradient elution method was employed with the mobile phases as follows: aqueous solution containing 0.06% formic acid (FA) and 10 mM ammonium formate at pH 3.6 (A), 0.1% FA in acetonitrile (B), and 0.1% FA in methanol (C). An adjustment to mobile phase A (MPA) preparation and storage was implemented to address shifts in pH over time. MPA was prepared in a 2,000mL glass bottle and split into five 400mL aliquots that were kept airtight with parafilm and stored at 4°C. At 40 hours an aliquot was allowed to adjust to room temperature for 6 hours and then used to replace the previous aliquot for MPA. A blank following by the pooled QC was ran to ensure no shift in retention time was observed. This process addressed the changes in pH due to the acidity in the core of the aqueous system increasing as the surface to volume ratio increased demonstrated in previous research (49). The total run time was 75 minutes with a flow rate of 50 µL/min and an injection volume of 1 µL.

The data collection was employed in full MS/MS2 with the following acquisition settings: spray voltage set at 4 kV (positive mode); capillary temperature maintained at 200°C; vaporizer temperature maintained at 33°C; sheath gas at 38 (arbitrary units); m/z range spanning from 100 to 1000; data acquisition in profile mode; 1 microscans; AGC target set at Standard; maximum injection time limited to 50 ms; mass resolution set at 120,000; RF Lens at 35%; HCD MS2 activation; fixed normalized collision energy, 10%; MS2 mass resolution set at 3000. To ensure instrument accuracy, calibration of the detector was performed before analysis, maintaining a mass tolerance of 5 ppm. Internal standards were employed to monitor retention time, ionization efficiency, and instrument stability throughout the analysis. The ultrahigh performance liquid chromatography-mass spectrometry (LC-MS) analytical platform was controlled by Xcalibur (version 3.1 SP1.6; Thermo). To monitor instrument stability, a pooled quality control (QC) was utilized.

### 2.6 Untargeted chiral metabolomic analysis data processing

Raw files underwent processing using MS-DIAL (RIKEN Center for Sustainable Resource Science, Yokohama City, Kanagawa, Japan) and a MATLAB script. Spectral alignment and peak picking were conducted within MS-DIAL, with integrated intensities, monoisotopic masses, and retention times exported for further analysis in the MATLAB programming environment. Probabilistic Quotient Normalization was applied to all data. For all features, one tailed t-tests as well as log2 fold change was calculated between IgH-aberration-positive and IgH-aberration-negative patients at diagnosis. Metabolite identifications were determined by matching accurate masses and retention times to a mass spectral metabolite library of standards (IROA 300, Mass Spectrometry Metabolite Library of Standards (MSMLS); IROA Technologies, Sea Girt, NJ, USA). Features with a M/Z difference less than 5ppm and a retention time difference of less than three minutes were considered potential enantiomers. Only those that displayed statistical significance in at least one form were evaluated further. Unknowns were categorized based on their average peak intensity, with enantiomer one (E1) having less abundance than enantiomer two (E2). Figures were generated using RStudio 2023.12.1.402 and R for Windows 4.3.1 programming platform (50, 51).

### 2.7 Next-generation sequencing

Total RNA was extracted from cells isolated from a subset of patients carrying an anomaly at the IgH locus using a Maxwell 16 Instrument (Promega Corporation) configured with the LEV (low elution volume) hardware. A LEV simply RNA Cells Kit was used according to the manufacturer’s protocol. Prior to sequencing, RNA quality was verified via BioAnalyzer 2100 (Agilent, Santa Clara, CA, USA). High-quality RNA was submitted to the Genomic Sequencing and Analysis Facility (GSAF) at the University of Texas at Austin for analysis. Library preparation was directional with polyA enrichment for the quantification of the coding transcriptome and detection of gene fusions. Sequencing was performed on an Illumina NovaSeq 6000 (Illumina Inc., San Diego, CA, USA). Analysis was performed in the BaseSpace Hub (Illumina). Gene names and TPM (transcripts per million) values for each sample were exported for evaluation.

### 2.8 Metabolomics and transcriptomics data integration with MetaboAnalyst

We retrieved RNA-seq sequencing datasets from Pediatric BCP-ALL patients without IgH aberrations from TARGET databases. Matched by age and gender, we carried out differential expression gene analysis between IgH-aberration-positive and -negative groups and identified differentially expressed genes (Adj. Pvalue<=1e-2 and fold change>=1.5). All raw RNA-sequencing datasets were processed by bcbio-nextgen Bulk RNA-seq pipeline and differential expression analysis was carried out by edgeR based on raw counts generated using htseq-count. The integration of -omics data was accomplished using Joint Pathway Analysis from MetaboAnalyst v5.0. Metabolomic data encompassed the fold changes between the IgH-aberration-positive and -negative groups at the time of diagnosis for all positively identified features. For transcriptomics, genes were included if the fold change exceeded 1.5 between the IgH-aberration-positive and -negative groups. The Hypergeometric test, degree centrality, and combine p values (unweighted) was selected for the enrichment analysis, topology measure, and integration method, respectively. Figures were generated using R programming platform (50).

### 2.9 D- enantiomer correlation with genes representative of and IgH aberration

The genes with a CV <0.25 and >0 were considered to ensure that expression levels were representative of samples carrying IgH aberrations and not patient-specific features. The metabolites that exhibited statistical significance (D-Glutamine, D-Asparagine, D-Leucine, D-Valine, D-Phenylalanine) through chiral metabolomics analysis were considered for the integration analysis. Further, the L-form of these amino acids was also considered for the correlation analysis. The gene expression levels were correlated with the metabolite (i.e., single metabolite) concentration values from 5 different IgH-aberration-positive patients based on Pearson’s correlation coefficient. The genes that exhibited positive correlation (i.e., Pearson correlation coefficient r ≥ 0.7) with metabolites were considered for further integration analysis. Due to the absence of D-amino acid-specific pathways in the KEGG database, enrichment analysis was not performed. Thus, the Wikipathway database was used to retrieve the D-amino acid involving pathways (52). Subsequently, the genes involved in those pathways were retrieved to check the commonality with the list of genes that showed a positive correlation with metabolites. Furthermore, the positively correlated genes were manually integrated against the D-amino acid involving pathways from the Wikipathway database.

## 3 Results

### 3.1 Clinical and demographic characteristics of BCP-ALL patients

In this study, we examined ten pediatric patients diagnosed with BCP-ALL, evenly split between those with and without an aberration at the IgH locus. Patient’s characteristics and demographics are reported in **Table 1**. Patients were matched to minimize differences between patients with an anomaly at the IgH locus and those without. Sex, age and weight were matched between groups while race and ethnicity classifications were similar between groups. The median age was 11 (interquartile range (IQR) = 4–17), there were more females than males (4 males, 6 females). Almost all patients identified as white aside from one patient who did not disclose race and 8 of the 10 patients reported their ethnicity as Hispanic or Latino. The Wilcoxon rank sum tests on age, weight, and BMI are reported in **Table 2**, and resulted in no significant difference for any of the characteristics. However, one patient was removed from the analysis due to severe adverse reaction to standard of care treatment. The Wilcoxon rank sum tests were recalculated without this patient and are reported in **Table 2**, there was no significance observed for any of the characteristics.

**Table 1:** Pediatric BCP-ALL patient characteristics and demographics.

Characteristic	All	IgH-aberration-negative	IgH-aberration-positive
*Age (years)*	11 (4–17)	11 (4–17)	11 (4–14)
*Sex*	4 Male	2 Male	2 Male
	6 Female	3 Female	3 Female
*Weight (kg)*	45.8 (18.8–71)	45.5 (18.8–62.6)	47.0 (26.5–70.5)
*BMI(kg/m^2^)*	19.4 (16.7–30.0)	19.4 (16.8–23.42)	20.3 (16.7–29.8)
*Race*	9 White	5 White	4 White
	1 Declined		1 Declined
*Ethnicity*	8 Hispanic	4 Hispanic	4 Hispanic
	2 Non-Hispanic	1 Non-Hispanic	1 Non-Hispanic
*Risk Stratification*	8 High/Very High	4 High/Very High	4 High/Very High
	2 Low/Standard	1 Low/Standard	1 Low/Standard

Patient characteristics and demographics were matched between groups. IgH, immunoglobulin heavy chain; BMI, body mass index.

**Table 2:** For each patient characteristic, the nonparametric Wilcoxon rank sum test was completed to observe any significance between the groups for each characteristic.

*Characteristic*	*Patient Included*		*Patient Excluded*	
	*W*	*p-value*	*W*	*p-value*
*Age (years)*	14	0.83	9	0.9
*Weight (kg)*	11	0.84	7	0.56
*BMI(kg/m^2^)*	12	1	8	0.73

One IgH-aberration-negative patient was removed from the analysis due to an adverse reaction to treatment.

The CBCC team established diagnoses, assessed risks, and devised treatment plans based on predefined clinical parameters outlined in the methodology section. Diagnosis primarily relied on immunophenotyping flow cytometry, with specific cell type counts, including blasts (**Supplementary Table S2**). The IgH-aberration-positive patients had a range of 88.7%-96.4% with an average of 92.2% blast cells while the IgH-aberration-negative patients had a range of 60%-94.8% with an average of 74.4% (p-value = 0.07, **Supplementary Table S2**). Risk assessment incorporated full blood panel results, including WBC, available in **Supplementary Table S3**. A two-tailed, unpaired t-test was employed to compare the groups across all parameters in **Supplementary Table S2** and **Supplementary Table S3**, with no significant differences observed.

The CBCC team also assessed the prognostic implications of cytogenetic fluorescence *in situ* hybridization results for each patient (**Supplementary Table S4**). Among the IgH-aberration-positive patients a translocation, two deletions, a trisomy, and a fusion were identified. One IgH-aberration-positive patient exhibited an IgH translocation at 14q32, which was considered associated with poor prognosis (**Supplementary Tables S4**, **S5**). One IgH-aberration-negative patient carried an aberration associated with a favorable prognosis, a ETV6/RUNX1 fusion, recognized by the WHO foundation (**Supplementary Table S4**). Prognosis association marked as “Unknown” indicate cytogenetic aberrations whose prognostic impact remained undetermined by the CBCC team, while “NA” denotes cases where cytogenetic results lacked any in depth discussion regarding prognosis (**Supplementary Table S4**). Specific clinical notes about the IgH-aberration-positive patients marked as “Unknown” can be found in **Supplementary Table S5**. Two patients were classified as low/standard risk and 8 were high/very high risk based on the clinical parameters described in **Table 1** (**Supplementary Table S4**). Each pair of matched patients was treated by the same COG protocol, described in the methods section.

### 3.2 Untargeted chiral metabolomics analysis by UPLC-MS at diagnosis

**Figure 1:**
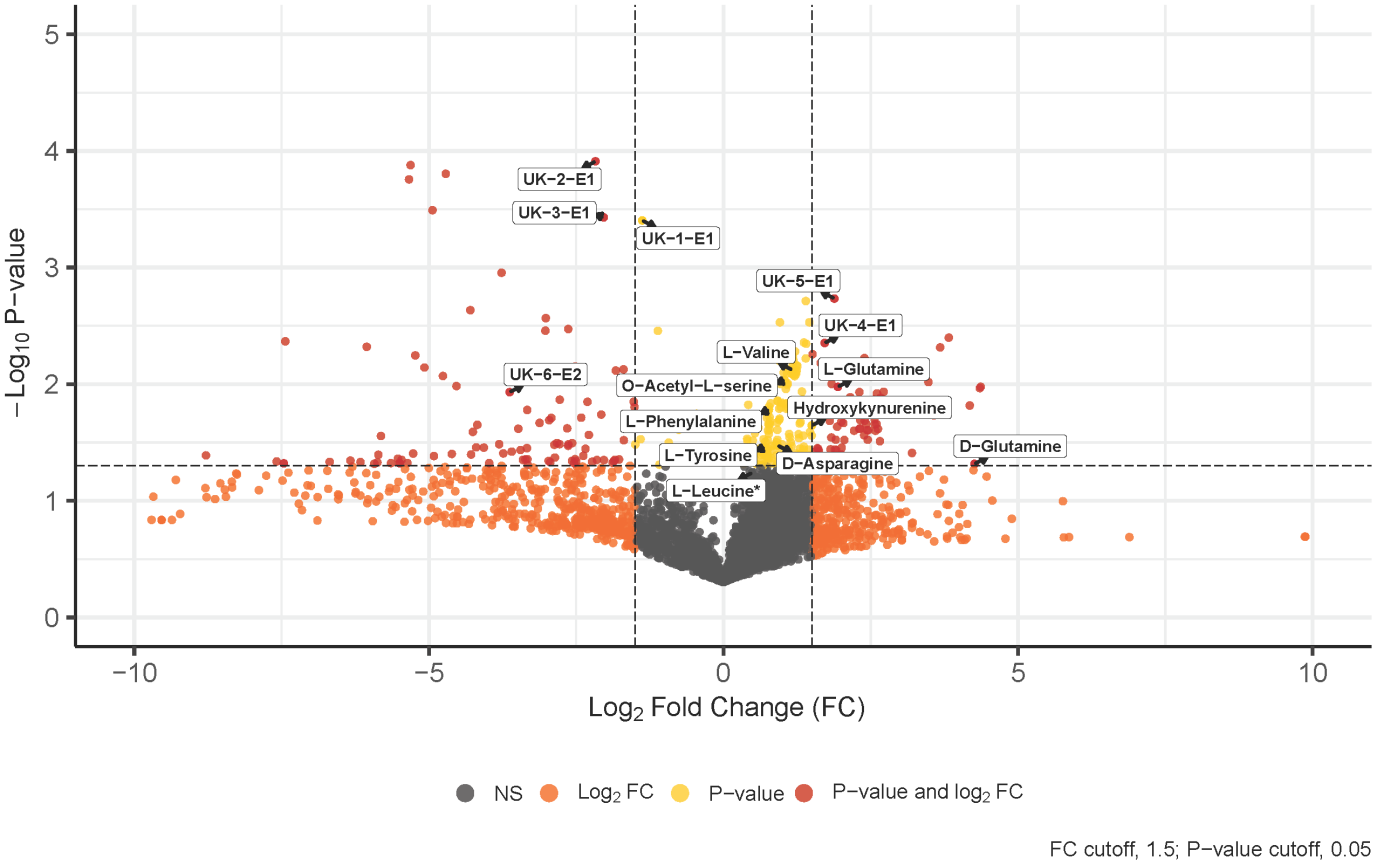
Volcano plot displays 4,500+ features detected by untargeted chiral metabolomics analysis of the intracellular metabolome at diagnosis. Annotated and unknown features exhibit significance, p-value < 0.05, in BCP-ALL pediatric patients with an aberration at the IgH locus’s versus those without. L-Leucine was considered borderline significant with a p-value between 0.05–0.06, denoted by an asterisk. BCP-ALL, B-cell precursor acute lymphoblastic leukemia; IgH, immunoglobulin heavy chain.

**Figure 2:**
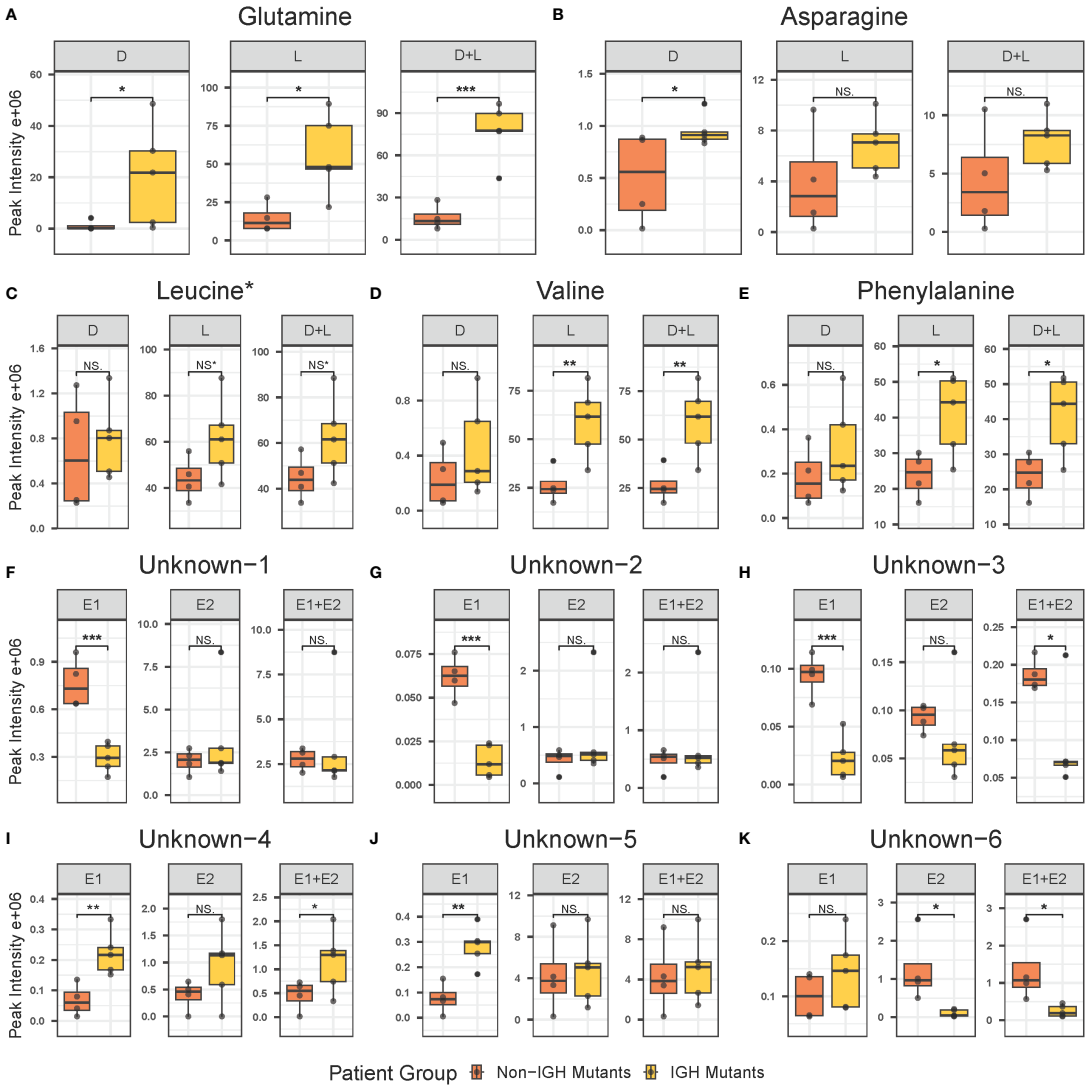
Individual box plots of annotated and unknown features from untargeted chiral metabolomics analysis exhibiting statistical significance in at least one form in the intracellular metabolome at diagnosis in BCP-ALL pediatric patients. Annotated features include glutamine **(A)**, asparagine **(B)**, leucine **(C)**, valine **(D)**, and phenylalanine **(E)**. Unknown features include unknown-1 **(F)**, unknown-2 **(G)**, unknown-3 **(H)**, unknown-4 **(I)**, unknown-5 **(J)**, and unknown-6 **(K)**. BCP-ALL, B-cell precursor acute lymphoblastic leukemia. If significance level is less than 0.001, “***”less than 0.01, “**”less than 0.05, “*”borderline significant 0.05–0.06, NS* not significant, NS. BCP-ALL, B-cell precursor acute lymphoblastic leukemia; IgH, immunoglobulin heavy chain.

Untargeted metabolomics analysis was performed on intracellular extracts of leukemia cells isolated from bone marrow aspirates collected from patients at the time of diagnosis. The total number of features resulting from the analysis was 4,884, with 359 of them being significant at diagnosis and 121 exhibiting a log2 fold change greater than 1.5 between the IgH-aberration-positive and -negative patient groups (**Figure 1**). In total 60 metabolites, 10 achiral and 50 chiral, were positively identified (**Supplementary Table S6**). Of these metabolites 8 exhibited a p-value < 0.05 between the two groups (**Figure 1**). Both forms of 14 enantiomeric metabolites including the amino acids’ glutamine, asparagine, leucine, valine, and phenylalanine were positively identified (**Figure 2**). Though all these amino acids were increased in the IgH-aberration-positive versus the IgH-aberration-negative group, glutamine was the only metabolite in which both the D- and L- forms as well as the total pool were statistically significant (**Figure 2A**). In the case of asparagine, only the D- enantiomeric form was significant while for valine and phenylalanine only the L- enantiomeric form was significant (**Figures 2B, E, F**). L-Leucine and the total pool of D + L-Leucine was considered borderline significant, exhibiting a p-value between 0.05–0.06 which is denoted by an asterisk (**Figures 1**, **2C**).

Additionally, six unknown features were determined to be potential enantiomeric pairs where at least one of the isomers displayed statistical significance between the two groups (**Figure 1**). The unknown-1 and 2 metabolites E1 forms exhibited a significant decrease while the E2 and E1 + E2 pool was unchanged between the two groups (**Figures 2F, G**). The unknown-3 metabolite exhibited a large decrease in both the E1 and E2 forms (**Figure 2H**). However, only the E1 form was significant, and the significance was largely decreased in the E1 + E2 pool (**Figure 2H**). Like the amino acids, the unknown-4 metabolite exhibited a large increase in both the E1 and E2 forms (**Figure 2I**). However, only the E1 form was significant, and the significance was largely decreased in the E1 + E2 pool (**Figure 2I**). The unknown-5 metabolite exhibited a significant increase in the E1 form while the E2 form was insignificant (**Figure 2J**). The unknown-6 metabolite exhibited a significant decrease in the E2 form while the E1 form was insignificant and as total pool, the change in significance was negligible (**Figure 2K**). The switching observed for unknown-1 in the pos, and neg-DATAN total pool quality controls (QCs) demonstrates the potential of an enantiomeric compound (**Supplementary Figure S1**).

### 3.3 Positively identified metabolites and transcriptomics KEGG joint pathway analysis at diagnosis

**Figure 3:**
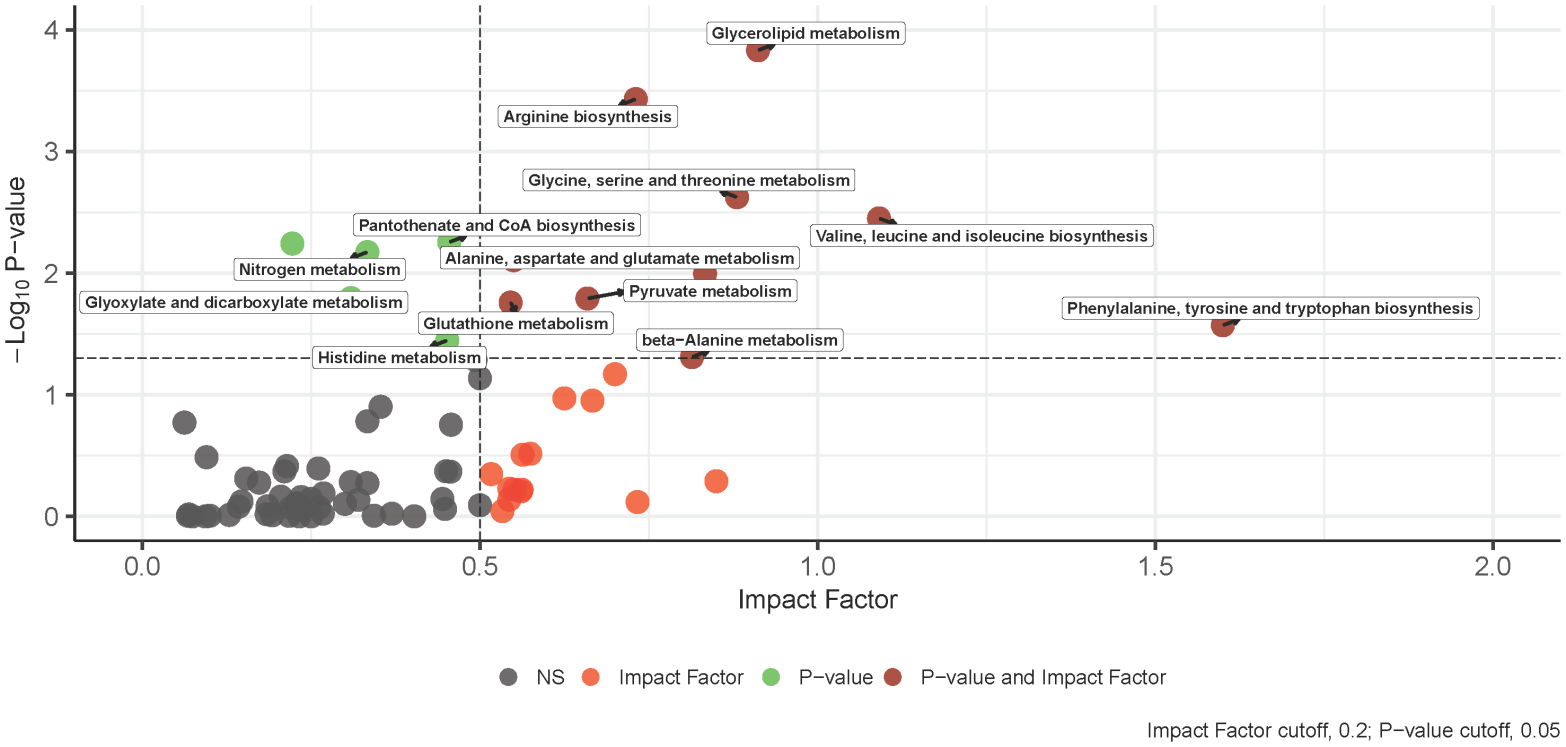
An overview of integrative pathway analysis by MetaboAnalyst. Pathway analysis was performed on UPLC‐MS features positively identified by accurate mass and retention time and genes from transcriptomics analysis. Only pathways with a p-value below 0.05 and an impact score of more than 0.2 were labeled. UPLC‐MS, ultra-performance liquid chromatography–mass spectrometry.

The integration of -omics data was accomplished using MetaboAnalyst v5.0 joint pathway analysis mapped to established KEGG metabolic pathways (53). A total of 79 pathways underwent evaluation, with 14 of them having both at least one compound and gene hit as well as demonstrating statistical significance (p-value < 0.05) and an impact factor greater than 0.2 (**Figure 3**). Detailed results, including raw values, are provided in **Table 3**. Among the sixteen pathways identified as significant based on their p-value and impact factor, ten are associated with amino and hydroxy acid metabolism or biosynthesis, while the remaining pathways are linked to oxidative stress and lipid metabolism. When examining the compounds and gene hits associated with each pathway it was revealed that glycine, serine, and threonine metabolism had several statistically significant genes (p-value < 0.05) between the two groups. The upregulation of PHGDH, PSAT1, and SARDH, and downregulation of ALDH7A1, GATM, and GLDC were observed. The positively identified metabolites of this pathway included D-glycerate, L-threonine, L-serine, D-serine, and glycine all of which were increased in the IgH-aberration-positive group, though none of them individually reached significance.

**Table 3:** MetaboAnalyst integrative pathway analysis raw results.

Pathway	Total	Hits.cmpd	Hits.gene	p-value	Impact
*Glycerolipid metabolism*	35	2	13	1.47E-04	0.9
*Arginine biosynthesis*	27	4	4	3.71E-04	0.7
*Glycine, serine and threonine metabolism*	68	5	8	2.38E-03	0.9
*Valine, leucine and isoleucine biosynthesis*	12	3	1	3.54E-03	1.1
*Pantothenate and CoA biosynthesis*	34	3	5	5.56E-03	0.5
*Nitrogen metabolism*	10	1	4	6.70E-03	0.3
*Alanine, aspartate and glutamate metabolism*	61	5	5	7.82E-03	0.6
*Glyoxylate and dicarboxylate metabolism*	56	5	3	1.60E-02	0.3
*Pyruvate metabolism*	45	3	6	1.62E-02	0.7
*Glutathione metabolism*	56	3	8	1.75E-02	0.5
*Phenylalanine, tyrosine and tryptophan biosynthesis*	11	2	1	2.68E-02	1.6
*Histidine metabolism*	32	2	5	3.57E-02	0.5
*beta-Alanine metabolism*	44	2	7	4.90E-02	0.8

Total denotes the potential count of hits associated with each pathway, encompassing both compounds and genes. The tally of overall hits derived from the input is recorded in the columns hits.cmpd and hits.genes. Only pathways with both a compound and gene hit as well as a p-value below 0.05 and an impact score greater than 0.2 are included.

### 3.4 D- enantiomer correlation with genes representative of IgH aberration at diagnosis

**Figure 4:**
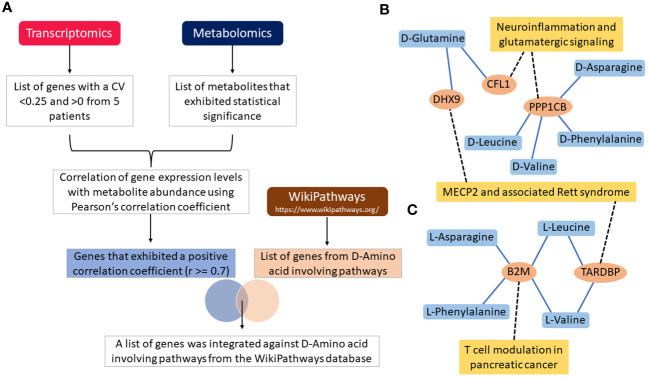
Flow chart illustration of transcriptomics and metabolomics data correlation and pathway integration **(A)**. Note that the DHX9, CFL1, PPP1CB, B2M and TARDBP are the overlapping genes between the positively correlated genes and D-Amino acid-involving pathways genes. Gene-metabolite interaction networks for the overlapping genes with **(B)** D-Amino acids, and **(C)** L-Amino acids. Only positively correlated genes that are involved in D-Amino acid-involving pathways are alone shown in the network. Note that the genes correlated with metabolites are shown in the blue colored lines and pathways integrated with genes are shown in black colored dotted lines.

Following filtering of transcriptomics genes described in the methods, 328 were considered representative of samples carrying IgH-aberrations. The correlation was determined between these genes and metabolites using Pearson’s correlation coefficient. In the five patients examined, a total of 32, 41, 23, 23, and 20 genes were positively correlated with D-asparagine, D-glutamine, D-leucine, D-phenylalanine, and D-valine, respectively, with correlation coefficient values of r≥0.7 (**Supplementary Table S7**). A total of 108 genes that were reported in D-amino acid-involving pathways within the Wikipathway database were retrieved and checked against positively correlated genes with the D-AAs to retrieve the overlapping genes for further integration analyses (**Figure 4A**). The analyses revealed 5 genes including CFL1, PPP1CB, B2M, TARDBP and DHX9, as overlapping genes. The genes CFL1 and DHX9 were shown to correlate with D-glutamine while PPP1CB was shown to correlate with D-asparagine, D-leucine, D-valine and D-phenylalanine. Further, these genes were reported to be involved in the neuroinflammation and glutamatergic signaling pathway within the Wikipathway database (**Figure 4B**) (52).

On the other hand, a total of 38, 20, 71, 22, and 42 genes were positively correlated with L-asparagine, L-glutamine, L-leucine, L-phenylalanine, and L-valine, respectively, with correlation coefficient value of r≥0.7 (**Supplementary Table S8** **).** The B2M gene was shown to correlate with L-asparagine, L-phenylalanine, L-valine, L-leucine. B2M is known to be involved in T-cell modulation in the pancreatic cancer pathway [38]. Genes such as TARDBP and DHX9 were shown to correlate with L-leucine and L-valine. Further, these genes were involved in MECP2 and associated Rett syndrome pathway (**Figure 4C**) (52). All overlapping genes were also found to be associated with poor prognosis and/or treatment resistance in other cancer types (**Figure 4B**) (52, 54–57).

## 4 Discussion

Despite the established knowledge that BCP-ALL pediatric patients with an IgH-aberration may face a worse prognosis; the treatment protocol has remained unchanged and is the same as for patients without an IgH-aberration. Russel et al. conducted an eight-year monitoring of pediatric ALL patients’ post-diagnosis, revealing a 40% decrease in survival rate and a 20% increase in relapse rate among those with an IgH translocation (18). However, the metabolic implications of such translocations or other IgH-aberrations in this population have not received thorough investigation. In recent years, abnormal concentrations of chiral metabolites have emerged as crucial indicators for disease monitoring and prognosis, guiding treatment decisions due to their association with pathological processes (58). Here, we present, for the first time, a comprehensive analysis of the intracellular chiral metabolome in leukemia cells from the bone marrow of pediatric BCP-ALL patients, comparing those with and without an IgH-aberration. This study demonstrates the efficacy of UCM as a robust platform, facilitating the simultaneous analysis of established, “unnatural,” and unknown chiral and achiral metabolites.

To the best of our knowledge, this is the first study to report the existence of D-amino acids within the intracellular metabolome of leukemia cells isolated from bone marrow aspirates. Despite the limited number of patients in our study, we demonstrate that IgH-aberration-positive patients share a distinct chiral metabolic phenotype. While our findings require further validation, they are significant because they identify both enantiomeric forms of metabolites, known to be crucial in cancer metabolism, as potential discriminators of patients carrying a class of specific genetic aberrations. Additionally, we unveiled enantiomeric metabolites of yet unknown potential whose importance would have been overlooked without the chiral separation.

The levels of several chiral metabolites were significantly altered in leukemia cells from newly diagnosed IgH-aberration-negative compared to IgH-aberration-positive patients. In patients with IgH aberrations, the levels of several L-amino acids, as well as D-Asn and D-Gln were increased. The heightened need for L-amino acids is a recognized trait of BCP-ALL cells, which rely on extracellular L-Asn and L-Gln for proliferation, and a metabolic vulnerability targeted in current chemotherapy strategies, which include PEG-L-ASP. This accumulation suggests that IgH-aberration-positive patients exhibit a phenotype less dependent on L-amino acid utilization compared to their counterparts, potentially explaining their reduced sensitivity to PEG-L-ASP treatment. D-Asn has been linked to breast cancer detection, while D-Gln metabolism has been associated with doxorubicin resistance in prostate cancer (59, 60). Additionally, while an increase in L amino acids may be linked to conventional pathways, the identification of D forms introduces avenues for deeper exploration. The detection of intracellular D-amino acids, notably D-Gln, which showed comparable abundance to L-Gln, raises fundamental questions. Firstly, their presence in the cell prompts inquiry into whether they were transported from the extracellular environment or if they were endogenously produced (61, 62). In both scenarios, the mechanism by which this occurs remains unknown, challenging our current understanding of amino acid transport and metabolism (61, 62). Whether it is a result of a gain of function in a transport protein or stems from an intrinsic mutation leading to the endogenous production of the unnatural enantiomer, analogous to D-2-hydroxyglutarate (2HG), either scenario could potentially impact the efficacy of treatment (40, 61–68).

Using matched metabolomic and transcriptomic samples for the IgH-aberration-positive patients, we employed two methods of data integration, ensuring our findings were aligned with established frameworks while also exploring the unknown metabolic features. In both approaches, only positively identified metabolites were included in the analysis. Given MetaboAnalyst maps to documented KEGG pathways and the relatively emergent nature of chiral metabolites in research, the several “unnatural” enantiomers included in the analysis were not recognized for incorporation within the platform. Despite this limitation, the obtained results remain pertinent as the metabolites that were successfully incorporated are still relevant and each of the identified pathways is dependent on chiral entities. Notably, this included a significant upregulation of PHGDH, PSAT1, and SARDH, alongside a significant downregulation of ALDH7A1, GATM, and GLDC. The detection of aberrant serine and glycine metabolism, which is present in a wide range of cancers and ALL cells, further underscores the significance of these findings (69–72). To address the constraint of mapping to the KEGG database, we utilized correlation analysis to reveal previously unknown associations of D and L-amino acids with genes specific to the IgH-aberration phenotype. This analysis unveiled a positive correlation between several D-amino acids and DHX9, CFL1, and PPP1CB, as well as a positive correlation between L-amino acids and B2M and TARDBP. Notably, each of these genes has previously been linked to poor prognosis and treatment resistance in other cancer types (54, 56, 73–78).

From the technical standpoint, the results are partially marred by the presence of features, that we have identified as likely enantiomeric forms that appear to have the potential of being biologically relevant metabolites which remained, as of this report, unidentified. In the development of UCM, a vast library of metabolite intermediates standards was utilized, meaning that the noteworthy unidentified features are likely metabolites that have not been extensively researched or are structurally unknown (37). This creates a common challenge arising from the limitation of unknown metabolites not being included in spectral databases and lacking chemical standards, making their validation a significant hurdle (79). In future analyses, we plan to overcome these limitations by utilizing IQ-X MS^n^ capabilities, which will aid in the identification of unknown metabolites.

### 4.1 Conclusions

Taken together, our results highlight the significance of utilizing UCM in conjunction with transcriptomics analysis to uncover unexplored pathways associated with the IgH-aberration-positive BCP-ALL chiral metabolic phenotype that may aid the improvement of treatment options. We have reported for the first time that D-amino acids are present within the intracellular metabolome of BCP-ALL cells isolated from the patients’ bone marrow. In tandem with IgH-aberration-positive patients exhibiting patterns and associations with several genes that have been previously linked to poor prognosis and/or treatment resistance, it is clear that the chiral metabolome may be playing a role in the patient’s outcome. Thus, our results underscore the need to develop innovative treatment strategies tailored to this subset of BCP-ALL patients while providing the foundational framework for future research endeavors that aim to explore the impacts of the chiral metabolome further.

## Data availability statement

Source data are provided with this paper and deposited in Zenodo open access repository (10.5281/zenodo.10937407).

## Ethics statement

The studies involving humans were approved by Children’s Blood and Cancer Center at the Dell Pediatric Medical Center in Austin, Texas. The studies were conducted in accordance with the local legislation and institutional requirements. Written informed consent for participation in this study was provided by the participants’ legal guardians/next of kin. Written informed consent was obtained from the minor(s)’ legal guardian/next of kin for the publication of any potentially identifiable images or data included in this article.

## Author contributions

MC: Conceptualization, Data curation, Formal analysis, Investigation, Methodology, Validation, Writing – original draft. RG: Formal analysis, Methodology, Writing – review & editing. VI: Formal analysis, Writing – review & editing. XP: Formal analysis, Methodology, Writing – review & editing. SC: Formal analysis, Methodology, Writing – review & editing. SY: Funding acquisition, Resources, Supervision, Writing – review & editing. AL: Supervision, Writing – review & editing. ST: Conceptualization, Funding acquisition, Supervision, Writing – review & editing.
