# Supplementary material for: Exploration of the intracellular chiral metabolome in pediatric BCP-ALL: a pilot study investigating the metabolic phenotype of IgH locus aberrations
Source: Front Oncol. 2024 Aug 5;14:1413264. doi: 10.3389/fonc.2024.1413264 (PMC11332069; doi:10.3389/fonc.2024.1413264)
Supplement: Supplementary file 1 [file DataSheet_1.pdf]

frontiers

Supplementary Material

Exploration of the Intracellular Chiral Metabolome in Pediatric BCP-ALL: A Pilot Study Investigating the Metabolic Phenotype of IGH Locus Aberrations

Meghan Collins[1,2], Ruggiero Gorgoglione[1,2], Valeria Impedovo[1,2], Xingxin Pan[3], Chakkarai Sathyaseelan[3], S. Stephen Yi[3,4,5], Alessia Lodi[1,2], Stefano Tiziani[1,2,3,6*]
[1]Department of Nutritional Sciences, College of Natural Sciences, The University of Texas at Austin, Austin, TX, 78712, USA.

[2]Dell Pediatric Research Institute, Dell Medical School, The University of Texas at Austin, Austin, TX, 78723, USA.

[3]Department of Oncology, Livestrong Cancer Institutes, Dell Medical School, The University of Texas at Austin, Austin, TX, 78723, USA.

[4]Interdisciplinary Life Sciences Graduate Programs, College of Natural Sciences, The University of Texas at Austin, Austin, TX, 78712, USA.

[5]Oden Institute for Computational Engineering & Sciences, and Department of Biomedical Engineering, The University of Texas at Austin, Austin, TX, 78712, USA.

[6]Department of Pediatrics, Dell Medical School, The University of Texas at Austin, Austin, TX, 78723, USA.

*** Correspondence:**
Stefano Tiziani, Ph.D.
tiziani@austin.utexas.edu

1 Supplementary Data

Supplementary Material should be uploaded separately on submission. Please include any supplementary data, figures and/or tables.

Supplementary material is not typeset so please ensure that all information is clearly presented, the appropriate caption is included in the file and not in the manuscript, and that the style conforms to the rest of the article.

2 Supplementary Figures and Tables

For more information on Supplementary Material and for details on the different file types accepted, please see here.

2.1 Supplementary Figures

Supplementary Figure 1. The figure legends are required to have the same font as the main text, 12 point normal Times New Roman, single spaced. Please use a single paragraph for each legend and prepare the figures keeping in mind the PDF layout.

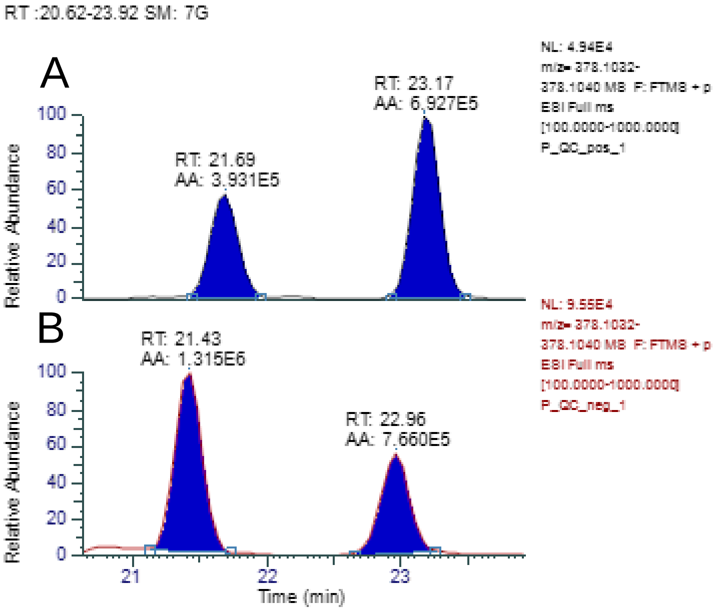

Figure S1. Chromatogram of unknown-1 at diagnosis derivatized with (+)-DATAN (**A**) and (-)-DATAN (**B**). Chiral switching can be observed

Table S1. Established parameters for clinical risk assessment of pediatric BCP-ALL patients.

Parameter	*Standard Risk*	*High Risk*
Age (years)	<10	≥10
Sex	Female	Male
WBC count (per µL)	<50,000	≥50,000
CNS infiltration	ND	D
MRD (Induction)	Negative	Positive
Cytogenetics	Hyperdiploid	Hypodiploid, Ph+, Ph-like, *MLL*

Table S2. Immunophenotyping flow cytometry results at diagnosis.

	Cell Percentages at Diagnosis						
Patient	*Viability*	*Lymphocytes*	*Monocytes*	*Granulocytes*	*Blasts*	*CD45-neg/Debris*	*Abnormal*
1-IGH	90.0%	2.6%	0.0%	0.4%	96.4%	0.2%	95.4%
2-IGH	95.0%	7.8%	0.1%	3.1%	88.7%	0.0%	89.0%
3-IGH	91.0%	10.0%	0.0%	0.2%	89.7%	0.0%	89.7%
4-IGH	99.0%	1.1%	0.1%	4.1%	94.1%	0.0%	94.6%
5-IGH	50.0%	5.2%	0.0%	2.2%	92.0%	10.0%	91.2%
6	92.0%	18.2%	3.1%	17.0%	60.0%	0.0%	60.0%
7	85.0%	3.8%	0.2%	10.7%	82.5%	1.4%	83.2%
8	88.0%	35.3%	0.1%	0.8%	60.1%	0.0%	62.2%
9	83.0%	1.8%	0.7%	NA	94.8%	NA	95.0%
P-Value	0.85	0.23	0.33	0.19	0.07	0.29	0.06

Table S3. Blood panel results.

Patient	*WBC K/ul*	*RBC (MIL)*	*HCT (%)*	*MCV (fl)*	*RDW (%)*	*PLT (K/ul)*
1-IGH	2.39	1.92	14.90	77.60	13.30	158.00
2-IGH	299.50	1.33	11.50	86.50	15.80	167.00
3-IGH	12.50	2.47	21.60	87.40	14.50	78.00
4-IGH	3.18	1.88	16.90	89.90	19.50	34.00
5-IGH	2.40	3.70	39.00	105.40	16.50	179.00
6	14.98	4.02	32.60	81.10	13.20	255.00
7	21.20	4.18	33.90	81.10	13.20	50.00
8	2.72	2.51	20.90	83.30	15.10	26.00
9	1.90	4.00	40.90	102.30	14.80	116.00
P-Value	0.45	0.04	0.13	0.73	0.19	0.84

Table S4. Cytogenetic results and prognosis association at diagnosis.

Patient	*Cytogenetics B-ALL FISH Results*	*Prognosis Association*	*Risk*
1-IGH	Three copies of lgH; no other abnormalities detected	Unknown	Standard
2-IGH	EPOR and IGH gene fusion	NA	High
3-IGH	IGH rearrangement (translocation at 14q32) and trisomy 21 and trisomy X in of nuclei	Unfavorable	Very High
4-IGH	ETV6 / RUNXI fusion detected, partial deletion of IgH region<br><br>No CRLF2/IgH fusion detected	NA	High
5-IGH	83% of nuclei had a deletion of the IGH variable region (at 14q32)<br><br>Indicates 10% of nuclei had a heterozygous CDKN2A deletion and 5% of nuclei had a homozygous CDKN2A deletion<br><br>No disruption of CRLF2 gene is detected	Unknown	Very High
6	Abnormal FISH with t (I; 19) PBX I : TCF 3 fusion detected in 52% of analyzed nuclei	NA	Standard
7	71.5% of nuclei have a homozygous CDKN2A deletion (at 9p21)	Unknown	High
9	ETV6/RUNX1 fusion detected in 64.2% of analyzed nuclei by FISH<br>No t(12;21) detected by karyotype, consistent with "cryptic" fusion	Favorable	High
10	90% of nulcei had trisomy 22, tetrasomy 10 and tetrasomy 21<br><br>92% of nuclei demonstrated an unbalanced disruption of the ABL2 gene region (at Iq25)<br><br>Above resulted in deletion of 3 i ABL2 gene region and a retention of the 5 ' ABL2 gene region on chromosome 10	Unknown	Very High

Table S5. Children's Blood and Cancer Center at the Dell Children's Medical Center cytogenetic prognosis of IGH patients at diagnosis.

Children's Blood and Cancer Center Cytogenetic Prognosis of IGH Patients

Patient	*Cytogenetics B-ALL FISH Prognosis Notes*
1-IGH	The morphologic and immunophenotypic findings are those of B-lymphoblastic leukemia. While three lgH copies are detected by FISH, including one with a deletion in the variable region, subsequent FISH interrogation showed no abnormalities involving genes associated with a so-called Ph-like ALL (normal signals for CRLF2, P2RY8, ABL2, PDGFRB, NUP98 and JAK2). The prognostic significance for this low level IGH abnormality in pediatric B-ALL is unknown (Moorman, Blood Reviews 26: 123—135, 2012)
3-IGH	Conventional cytogenetic analysis of bone marrow aspirate performed at Mayo Medical Laboratories (MI 34207507) revealed a t (10; 14), trisomy 21, and trisomy X in all metaphase cells. Fluorescence in situ hybridization (FISH) of bone marrow aspirate performed at Mayo Medical Laboratories (MI 34207507) revealed an IGH rearrangement (translocation at 14q32), and trisomy 21 and trisomy X in approximately 93% of nuclei. According to the outside report, at diagnosis, an IGH translocation is associated with an intermediate to unfavorable prognosis in pediatric patients with B—ALL. Please refer to the outside report for additional information.
5-IGH	The result is abnormal and indicates 10% of nuclei had a heterozygous CDKN2A deletion and 5% of nuclei had a homozygous CDKN2A deletion (at 9p21). In addition, 83 % of nuclei had a deletion of the IGH variable region (at 14q32) which could represent clonal expansion of a normal IGH variant or an unbalanced IGH translocation. Chromosome studies are normal (reported separately). At diagnosis, the prognostic significance for this clone in pediatric patients with B—ALL is unknown (Moorman, Blood Reviews 26: 123-135, 2012). No disruption of CRLF2 gene is detected.

Table S6. Positively identified metabolites at diagnosis.

Metabolite	Average m/z	Average RT(min)	Diagnosis								
			Patient ID								
			8	7	9	6	1	3	2	5	4
1-Aminocyclopropanecarboxylic acid	318.083	9.672	9.00E+04	1.51E+06	8.45E+05	1.02E+06	3.58E+05	1.24E+06	1.85E+06	7.79E+05	1.10E+06
2-Methoxyethanol	310.113	24.687	1.24E+05	5.79E+04	4.25E+04	7.87E+04	1.25E+05	5.84E+04	7.33E+04	1.20E+05	2.55E+04
3-Methyladenine	366.104	12.475	6.20E+03	2.84E+05	2.56E+05	1.99E+04	1.29E+04	2.78E+05	3.40E+05	2.09E+04	2.28E+05
3-Methylhistamine	342.13	5.562	1.03E+00	1.00E+00	1.00E+00	4.80E+04	1.00E+00	1.00E+00	1.00E+00	8.53E-01	1.00E+00
5-Aminopentanoic acid	334.113	24.054	1.04E+05	1.01E+05	2.34E+05	1.55E+05	1.08E+05	7.71E+04	4.20E+05	1.55E+05	3.79E+05
Alpha-Hydroxyisobutyric acid	338.108	35.06	2.41E+04	7.88E+04	2.14E+05	2.25E+05	5.90E+04	7.50E+05	8.39E+04	9.00E+04	1.88E+05
Beta-Alanine	306.082	7.22	1.76E+05	3.63E+05	3.09E+05	5.11E+05	5.74E+05	3.62E+05	4.16E+05	6.88E+05	2.95E+05
D-2-Hydroxy-3-methylbutyric acid	352.124	48.764	1.82E+05	1.07E+05	1.76E+05	1.13E+05	2.69E+05	1.80E+05	1.71E+05	1.73E+05	1.68E+05
D-2-Hydroxybutyric acid	338.108	32.529	1.12E+05	1.52E+05	1.05E+05	5.58E+04	9.82E+04	9.64E+04	1.23E+05	6.91E+04	1.31E+05
D-2-Hydroxyglutaric acid	382.098	15.93	2.69E+05	2.46E+06	9.79E+05	1.09E+06	3.45E+05	9.12E+05	1.56E+06	2.98E+05	7.63E+05
D-3-Hydroxykynurenine	674.167	46.399	1.03E+00	4.46E+04	1.43E+04	3.92E+03	6.23E+03	5.56E+03	7.42E+04	3.80E+04	2.22E+04
D-Alanine	306.082	9.155	1.31E+06	1.63E+07	1.52E+07	6.78E+06	3.41E+06	1.52E+07	1.52E+07	6.84E+06	1.83E+07
D-Asparagine	349.088	26.959	1.50E+04	8.65E+05	2.51E+05	8.86E+05	1.21E+06	9.12E+05	8.71E+05	8.33E+05	9.39E+05
D-Aspartic acid	350.072	7.72	2.02E+05	2.01E+05	2.00E+05	1.39E+06	1.62E+06	1.87E+05	3.16E+04	1.94E+06	3.09E+05
D-Carnitine	378.14	8.803	1.15E+07	1.60E+05	8.05E+04	1.36E+05	2.90E+07	3.20E+05	6.60E+05	9.06E+04	1.06E+05
D-Glutamine	363.104	6.184	4.14E+06	9.45E+04	2.28E+04	6.62E+04	3.03E+07	2.46E+06	3.83E+05	2.18E+07	4.86E+07
D-Glyceric acid	340.088	7.626	3.69E+04	3.42E+04	2.10E+04	1.51E+05	6.03E+04	1.07E+04	1.28E+05	2.16E+05	7.73E+04
D-Lactic acid	324.093	17.903	3.24E+06	8.26E+06	1.66E+07	1.28E+07	1.07E+07	1.98E+07	1.34E+07	1.41E+07	6.57E+06
D-Leucic acid	366.139	57.518	1.41E+05	9.48E+04	1.10E+05	4.13E+04	5.76E+04	7.88E+04	9.93E+04	4.36E+04	1.41E+05
D-Leucine	348.129	51.508	9.53E+05	2.29E+05	2.54E+05	1.27E+06	1.34E+06	4.56E+05	5.07E+05	8.04E+05	8.72E+05
D-Mevalonic acid	382.135	50.508	2.24E+05	1.34E+05	2.32E+05	6.77E+04	8.28E+04	1.99E+05	3.18E+05	6.52E+04	1.70E+05
D-Phenylalanine	382.114	55.455	2.14E+05	6.89E+04	9.70E+04	3.62E+05	6.31E+05	1.24E+05	2.35E+05	4.21E+05	1.71E+05
D-Serine	538.105	18.205	1.29E+05	8.25E+05	2.75E+05	6.53E+05	3.66E+05	7.14E+05	7.60E+05	3.55E+05	5.82E+05
D-Tryptophan	421.124	58.396	1.03E+00	3.45E+04	7.64E+05	2.28E+04	2.75E+04	1.00E+05	1.31E+06	3.59E+04	9.70E+04
D-Valine	334.114	32.833	3.02E+05	5.70E+04	7.45E+04	4.94E+05	9.63E+05	1.39E+05	2.05E+05	6.49E+05	2.88E+05
Glyceraldehyde	324.093	5.195	2.13E+05	2.12E+05	4.95E+05	3.23E+05	4.49E+04	3.87E+05	7.74E+05	4.42E+05	3.08E+05
Glycerol	542.136	30.766	7.00E+05	1.65E+07	2.82E+06	1.16E+06	1.42E+06	5.59E+06	2.80E+06	1.22E+06	1.77E+06
Glycine	292.066	5.742	1.30E+07	1.36E+08	1.00E+08	5.34E+07	4.81E+07	1.07E+08	1.23E+08	6.96E+07	1.27E+08

Glycolic acid	310.077	8.314	9.12E+05	1.34E+06	1.00E+06	1.03E+06	1.10E+06	9.53E+05	1.02E+06	1.55E+06	1.17E+06
Histamine	328.114	5.489	1.03E+00	3.47E+05	8.41E+04	4.75E+05	7.81E+04	1.00E+00	8.96E+05	8.53E-01	1.00E+00
Hydroxykynurenine	674.167	39.487	1.03E+00	1.50E+04	4.26E+04	5.93E+04	9.54E+04	5.30E+04	8.04E+04	1.37E+05	4.65E+04
L-2-Hydroxyglutaric acid	382.098	12.573	7.32E+05	2.72E+06	1.86E+06	9.29E+05	5.05E+05	1.08E+06	3.41E+06	3.33E+05	2.05E+06
L-3-Hydroxykynurenine	674.167	45.366	1.03E+00	3.68E+04	7.88E+04	3.55E+03	1.62E+05	1.00E+00	3.44E+04	1.99E+05	2.76E+04
L-Alanine	306.082	10.419	2.78E+07	2.81E+08	1.25E+08	1.58E+08	5.96E+07	1.88E+08	2.69E+08	1.20E+08	1.75E+08
L-Asparagine	349.088	28.858	2.78E+05	9.64E+06	1.54E+06	4.14E+06	7.06E+06	4.38E+06	1.01E+07	5.04E+06	7.75E+06
L-Aspartic acid	350.072	6.051	1.90E+06	4.59E+07	1.05E+07	1.97E+07	2.62E+07	2.81E+07	8.39E+07	1.94E+07	5.33E+07
L-Citrulline	392.131	6.999	3.75E+04	5.29E+05	4.19E+05	6.81E+05	1.41E+05	5.10E+05	7.18E+05	3.32E+05	4.30E+05
L-Glutamic acid	364.088	8.217	1.57E+08	7.60E+08	4.70E+08	3.37E+08	3.33E+08	8.25E+08	9.82E+08	4.66E+08	9.97E+08
L-Glutamine	363.104	5.506	7.77E+06	2.82E+07	7.91E+06	1.47E+07	4.68E+07	7.50E+07	8.94E+07	2.18E+07	4.80E+07
L-Glyceric acid	340.088	6.362	1.61E+07	6.92E+05	2.10E+06	2.88E+07	3.24E+07	3.97E+06	5.68E+05	4.15E+07	9.78E+05
L-Lactic acid	324.093	16.516	3.77E+07	3.12E+08	5.49E+08	2.19E+08	7.84E+07	6.29E+08	4.13E+08	3.08E+08	1.28E+08
L-Leucic acid	366.139	54.8	2.85E+05	2.13E+05	2.11E+05	1.40E+05	2.70E+05	1.90E+05	2.24E+05	2.75E+05	2.69E+05
L-Leucine	348.129	50.67	4.60E+07	3.36E+07	4.06E+07	5.59E+07	6.72E+07	5.08E+07	6.11E+07	4.16E+07	8.76E+07
L-Lysine	579.168	20.397	9.14E+06	1.75E+07	2.43E+07	2.39E+07	1.51E+07	2.80E+07	5.61E+07	2.23E+07	1.34E+07
L-Malic acid	368.083	7.796	3.44E+06	3.59E+07	1.79E+07	1.36E+07	5.79E+06	2.58E+07	1.47E+07	6.20E+06	1.98E+07
L-Methionine	366.085	31.794	1.45E+06	6.08E+06	1.25E+07	5.22E+06	5.75E+05	1.08E+07	1.93E+07	1.25E+06	1.19E+07
L-Ornithine	565.152	13.326	1.64E+06	2.07E+06	2.14E+06	3.18E+06	4.91E+06	2.31E+06	4.91E+06	3.25E+06	1.98E+06
L-Phenylalanine	382.113	54.093	2.16E+07	1.61E+07	2.77E+07	3.01E+07	5.11E+07	2.54E+07	5.03E+07	3.26E+07	4.42E+07
L-Serine	538.105	19.249	1.50E+07	1.99E+07	1.62E+07	3.43E+07	2.31E+07	2.76E+07	7.24E+07	1.43E+07	3.03E+07
L-Threonine	569.147	31.241	6.26E+06	1.61E+07	1.46E+07	2.18E+07	1.34E+07	2.96E+07	3.83E+07	2.06E+07	1.53E+07
L-Tyrosine	398.109	35.757	8.76E+06	8.56E+06	1.14E+07	1.00E+07	1.48E+07	1.04E+07	2.40E+07	1.17E+07	1.76E+07
L-Valine	334.114	33.65	2.38E+07	1.73E+07	2.48E+07	3.88E+07	6.89E+07	3.41E+07	6.17E+07	4.75E+07	8.15E+07
N-Acetyl-D-serine	364.088	16.378	4.53E+05	4.41E+06	2.11E+06	1.30E+06	2.13E+06	3.14E+06	4.65E+06	1.41E+06	3.27E+06
N-Acetyl-L-serine	364.088	12.275	1.19E+06	2.39E+06	2.68E+06	1.57E+06	1.36E+06	1.95E+06	1.30E+07	6.88E+05	3.07E+06
N-Acetylputrescine	347.145	19.896	7.06E+04	2.73E+06	1.31E+06	1.04E+06	4.00E+05	3.86E+06	9.63E+05	3.14E+05	2.21E+06
O-Acetyl-L-serine	364.088	13.648	3.50E+05	3.54E+05	3.00E+05	3.51E+05	9.07E+05	6.23E+05	9.60E+05	5.64E+05	4.24E+05
Putrescine	521.162	19.924	4.79E+05	1.92E+07	1.26E+07	6.84E+06	1.31E+06	2.07E+07	7.97E+06	2.86E+06	1.50E+07
Taurine	342.05	4.615	1.24E+07	2.20E+08	3.30E+07	1.08E+08	4.61E+07	1.15E+08	2.12E+08	6.77E+07	2.04E+08
UK-1-E1	378.104	22.783	9.60E+05	8.24E+05	6.36E+05	6.36E+05	3.68E+05	1.72E+05	3.96E+05	2.94E+05	2.39E+05
UK-1-E2	378.103	21.211	1.05E+06	2.31E+06	2.73E+06	1.83E+06	1.40E+06	2.73E+06	8.35E+06	1.84E+06	1.88E+06
UK-2-E1	416.06	22.631	7.60E+04	5.98E+04	6.51E+04	4.69E+04	2.27E+04	5.70E+03	2.39E+04	1.19E+04	4.53E+03
UK-2-E2	416.06	21.24	1.12E+05	5.01E+05	5.89E+05	4.68E+05	4.10E+05	5.52E+05	2.33E+06	5.12E+05	3.53E+05
UK-3-E1	423.162	22.781	9.91E+04	1.14E+05	6.90E+04	9.52E+04	2.03E+04	8.40E+03	5.24E+04	2.78E+04	6.39E+03

UK-3-E2	423.162	25.636	8.83E+04	1.02E+05	1.05E+05	7.39E+04	3.06E+04	5.84E+04	1.60E+05	4.36E+04	6.47E+04
UK-4-E1	993.233	8.473	1.44E+04	7.96E+04	1.35E+05	4.09E+04	3.33E+05	2.41E+05	2.17E+05	1.52E+05	1.68E+05
UK-4-E2	993.233	7.894	1.03E+00	6.45E+05	5.06E+05	4.10E+05	1.00E+00	1.80E+06	1.17E+06	5.89E+05	1.13E+06
UK-5-E1	305.098	50.498	3.09E+05	9.12E+06	4.15E+06	3.34E+06	1.17E+06	5.43E+06	9.68E+06	2.28E+06	5.06E+06
UK-5-E2	305.099	7.244	5.08E+03	8.12E+04	1.55E+05	6.68E+04	2.54E+05	2.99E+05	3.04E+05	3.90E+05	1.72E+05
UK-6-E1	437.15	62.285	6.57E+04	1.40E+05	1.34E+05	6.34E+04	7.94E+04	2.40E+05	1.46E+05	8.06E+04	1.74E+05
UK-6-E2	437.15	63.317	9.28E+05	2.56E+06	1.01E+06	5.04E+05	2.13E+04	2.16E+05	4.38E+04	3.60E+04	1.89E+05

Table S7. Pearson correlation of D- amino acids with IGH representative genes.

Gene	D-Asparagine	D-Glutamine	D-Leucine	D-Phenylalanine	D-Valine
CD74	-0.546169374	0.33017221	-0.376552556	-0.672813214	-0.625430108
TPT1	-0.27774289	-0.965401222	-0.582601218	-0.138938357	-0.318151083
B2M	0.645008909	0.364538802	0.569218001	0.392619506	0.334741458
PTMA	0.533319604	0.551093361	0.757051655	0.671183043	0.794056724
HNRNPA1	-0.076725549	-0.225926356	0.18832881	0.605053996	0.582630763
HLA-B	-0.620900606	-0.385803621	-0.869944948	-0.974622142	-0.995815336
HNRNPH1	-0.627782405	0.444797279	-0.197717837	-0.372471912	-0.297623033
RPS15A	0.193288119	-0.536615049	0.166086345	0.657634251	0.545343901
NACA	-0.100312045	-0.522375235	0.006256978	0.517954742	0.437217136
PPIA	0.783578073	-0.023871334	0.547289971	0.597090798	0.607273951
HLA-A	0.020259339	0.151653469	-0.035414557	-0.207311923	-0.063001677
NPM1	0.286556302	-0.370617982	0.263533338	0.648988504	0.608934122
HMGB1	-0.801537094	-0.523102821	-0.888962645	-0.7816828	-0.764676657
HLA-DPB1	-0.187231091	0.0679502	0.142645006	0.332769431	0.246905384
HLA-DPA1	-0.355788891	0.08215865	-0.273989012	-0.382709761	-0.445384245
HNRNPA2B1	0.288309018	0.843458276	0.3620425	-0.192260372	-0.04715999
MYL6	-0.035097866	-0.014049616	0.337479029	0.688233052	0.679673176
UBB	-0.020006059	0.646871583	0.227667982	-0.096882151	-0.063109493
PCBP2	-0.840272957	0.00923841	-0.529658864	-0.512945071	-0.419768962
EEF1D	0.069548026	-0.357795741	0.226242956	0.699260487	0.622521215
GNAS	-0.232086878	0.735194836	0.01129632	-0.457864077	-0.302100163
RPS13	0.145256914	-0.556263285	0.121876031	0.618562833	0.514823842
FAU	0.037893806	-0.650252257	-0.010877217	0.506334741	0.343872588
HLA-E	-0.091162905	0.818958797	0.447427498	0.229019905	0.362828514
OAZ1	0.330057176	0.565912399	0.251825629	-0.19778143	-0.165433923
SRSF5	-0.779997019	-0.341344625	-0.545160606	-0.285449792	-0.373188776
CFL1	0.572940873	0.81990465	0.56017278	0.035461796	0.159614842
SF1	-0.924114218	-0.481517784	-0.779958309	-0.531827476	-0.605074886
FTL	0.192787366	-0.189979829	0.400525602	0.817951834	0.742564778
LENG8	-0.287095312	0.694248442	0.273132421	0.118118094	0.231318789
EIF4A2	-0.627001026	-0.847934667	-0.732165071	-0.312526007	-0.471106301

AES	0.107223299	-0.045164417	0.32485737	0.603242307	0.64269382
HNRNPK	-0.560873097	0.446298371	-0.315545186	-0.642063955	-0.492134088
HNRNPC	-0.030874613	0.607594671	-0.01390948	-0.523890089	-0.427573255
ALDOA	0.531316156	-0.105061092	0.128411945	0.010647106	0.052310993
TPM3	-0.821374024	-0.344036949	-0.690428284	-0.539183371	-0.619613947
HNRNPDL	0.520533662	0.839853604	0.618708172	0.165540035	0.337153604
COX4I1	0.118618825	-0.642038961	-0.088165044	0.2986535	0.103349117
PFN1	0.640550588	-0.286934256	0.214766407	0.264226476	0.243367829
HNRNPD	0.127691583	0.660492654	0.119801798	-0.406273985	-0.242822738
NCL	-0.072158165	-0.053329955	-0.035139014	0.024423555	0.130921171
HNRNPU	-0.052538826	0.567241812	-0.048030854	-0.540722944	-0.377167458
YBX1	0.242322893	-0.046679514	0.02685331	-0.055994395	0.038935937
NPIPB5	0.650513687	0.87010477	0.769414991	0.349557268	0.430598979
SERF2	0.906346322	0.5126426	0.692773591	0.346588736	0.395355751
CIRBP	-0.125991582	0.717288045	0.242169879	-0.062506015	0.000589923
CYBA	0.753283541	0.715005114	0.649527513	0.185799631	0.274840661
SRSF11	-0.720227205	-0.209318468	-0.604163402	-0.549201694	-0.622345655
EIF3F	-0.24536514	-0.500216982	-0.684773445	-0.749995239	-0.755028904
GMFG	-0.031035542	0.329402682	-0.211724592	-0.669739335	-0.549548704
NAP1L1	0.21394436	-0.433943489	-0.066514233	0.108804046	0.118502956
EIF4G2	-0.817994608	0.093564099	-0.395281591	-0.339626363	-0.319230959
EIF3L	-0.402468473	-0.54166071	-0.208367426	0.305173311	0.225585368
DDX39B	0.352357179	0.69656945	0.30406186	-0.22050788	-0.136206755
CCNI	-0.307256596	-0.903004579	-0.578492245	-0.166114926	-0.354418545
EIF4B	-0.308346233	-0.882724915	-0.434705087	0.096597091	-0.07472427
YWHAZ	-0.549198868	0.07479951	-0.301678487	-0.310099342	-0.360512472
CSDE1	-0.40003609	-0.320027121	-0.300551997	-0.065602752	-0.206745751
PFDN5	0.108572755	-0.60908528	-0.001738882	0.444681161	0.260175538
MATR3	-0.719665975	0.363026931	-0.387977319	-0.614358263	-0.514233604
RBMX	-0.473759643	0.45787956	-0.187799129	-0.45755406	-0.285367024
ATP5G2	0.491521881	-0.337142887	0.311459929	0.586728185	0.557281771
PSMA6	-0.388194158	-0.066556741	-0.582232628	-0.83022943	-0.736135898
MORF4L1	-0.405824057	0.657000715	0.076900208	-0.157604033	-0.058671663
DAZAP2	-0.79232133	-0.462163885	-0.580817229	-0.244164762	-0.341559559
HSP90AA1	-0.70250058	-0.827088773	-0.715715286	-0.250158498	-0.343693514
MYH9	-0.226764709	-0.057635496	-0.495691767	-0.772605887	-0.79669787
SLC25A3	-0.333541438	-0.419241173	-0.082315085	0.40200841	0.29556829
ATP5B	0.316178546	-0.702602753	-0.183946056	0.044397387	-0.052159676
PSME1	0.514186068	-0.108924907	0.328827831	0.409189512	0.267648149
EWSR1	-0.047249763	0.803890822	0.205882364	-0.252608859	-0.061145962
SET	0.315966274	0.513624006	0.216518357	-0.210018292	-0.048603641
CALM1	-0.137533536	-0.911558015	-0.4350066	0.01439855	-0.175672586
CD19	0.940995473	0.656166321	0.911954061	0.628225216	0.6899249

WIPF1	-0.045501202	0.663674393	0.513916147	0.461561216	0.568477288
CD79B	0.953407312	0.433388988	0.728918041	0.455018672	0.497529684
RBM5	-0.003832611	0.356607579	0.114306246	-0.080971557	-0.122687844
HNRNPA3	0.188747787	0.803132703	0.327377681	-0.162448986	0.027154825
PABPN1	0.058437972	0.617574328	0.029060122	-0.503179902	-0.355480056
HNRNPM	-0.254831547	0.70987492	0.022380831	-0.400096115	-0.218680486
MAN2C1	0.777042351	0.171989644	0.815492457	0.953973613	0.945332923
SPPL2B	0.031121502	-0.647714612	-0.350093702	-0.14662146	-0.173057463
TMEM259	-0.616061162	0.210567202	-0.422071305	-0.616625069	-0.604317958
C19orf43	0.924681242	0.31783075	0.887238636	0.877816125	0.889851411
GABARAP	0.638679627	0.632254335	0.521616226	0.078140094	0.120596937
HNRNPUL1	-0.649530594	0.402524715	-0.243380452	-0.410038308	-0.269393676
TKT	-0.554864837	0.432588485	-0.333748847	-0.670814177	-0.582746822
CNBP	0.034395896	-0.015727861	-0.25609404	-0.502415408	-0.401612701
ARPC2	-0.2459265	-0.393221244	-0.227977251	0.041665709	-0.1222805
COX7C	0.709170511	-0.257344557	0.429484823	0.610445155	0.475120698
EDF1	0.698027815	0.047092635	0.415163712	0.334176134	0.386426539
ACIN1	0.519611039	0.916588905	0.626344814	0.11449132	0.242692837
TMA7	0.495647074	-0.564077134	-0.042652076	0.089036632	-0.015608415
EIF5A	-0.302158904	-0.867040465	-0.445433725	0.0583126	-0.12452645
USF2	-0.189952361	0.424971615	-0.247572239	-0.722212725	-0.594561551
CLIC1	0.431033501	-0.426305708	0.340411725	0.75398299	0.618302417
HNRNPH3	-0.465802022	0.371465108	-0.389274699	-0.781566414	-0.693953554
U2AF1	-0.575731391	-0.437583542	-0.577998344	-0.390492408	-0.343052195
ARPC3	0.47180727	-0.170669231	0.124166138	0.091321225	-0.038339227
EIF4H	-0.782655153	-0.244836746	-0.573681413	-0.417961356	-0.352563571
DCAF8	-0.065107018	0.717538383	0.091782691	-0.3913384	-0.291482425
ARF1	-0.706717716	-0.814694761	-0.691673903	-0.212045339	-0.330539067
BAG6	0.405655862	0.883788044	0.561394888	0.088233289	0.269195141
EIF3G	0.336555435	-0.439521325	0.295109868	0.73680228	0.641830417
CHD4	0.591446414	0.408319353	0.820484881	0.878694769	0.943350477
EEF1A1P5	0.281345193	-0.482455287	0.209809757	0.640573251	0.477261995
CD37	0.624761804	0.335941953	0.643636879	0.57756159	0.679699899
RAP1B	-0.303581956	0.412780205	-0.152506491	-0.459486267	-0.440237629
CDC37	-0.033319861	0.622540326	-0.012931351	-0.531855783	-0.375342633
SMG1P1	0.393003185	0.808077227	0.832261287	0.683860059	0.765332919
PRKCSH	0.328499947	0.590287851	0.235747518	-0.245913103	-0.086749967
SRSF6	-0.357341129	0.019894106	0.10489619	0.408240704	0.426694976
NDUFV1	0.527568726	0.552422682	0.319526441	-0.175456468	-0.06433244
PNN	-0.524944656	0.547347207	-0.163740551	-0.457260587	-0.362893226
TMBIM6	-0.402211319	-0.05470718	-0.630919501	-0.927702622	-0.903688243
SKP1	-0.052104593	-0.754139946	-0.585116289	-0.462569777	-0.59067888
CD81	0.347279666	0.748204588	0.686403694	0.482127415	0.644953825

ATP5A1	0.953509771	0.20338065	0.63137307	0.460703181	0.450980234
CHCHD2	0.5999931	-0.38815556	0.267351871	0.477042097	0.427714694
PSMB4	-0.089974815	-0.790704445	-0.423750454	-0.096395467	-0.291017361
ZNF207	-0.212862284	0.503616039	0.108519303	-0.087008591	-0.079159098
U2AF2	0.635468158	0.678718424	0.514481776	0.028584618	0.155682569
TCP1	-0.764580602	-0.608434624	-0.650220741	-0.269608128	-0.295609105
ST13	0.50333557	-0.39792746	0.244713393	0.501566276	0.463296178
UBAP2L	-0.247464826	0.417825951	-0.23953406	-0.664234045	-0.506938391
GDI2	0.283256867	-0.738920699	-0.189586989	0.083155834	-0.086404248
MRFAP1	0.245107006	-0.52553645	-0.237588365	-0.165014044	-0.188519267
SNHG1	-0.105375688	0.471817553	0.055119841	-0.230207222	-0.22945715
PAX5	-0.18517453	0.193033748	-0.257422049	-0.549887196	-0.57284006
SRP14	-0.228375577	-0.388848249	-0.663925178	-0.815891202	-0.857593904
SASH3	-0.277440153	-0.489164977	-0.678427633	-0.729301146	-0.824592161
ARL6IP4	0.684123664	0.112083071	0.239955037	-0.053416678	-0.039306605
CPNE1	0.613913885	0.20945665	0.280362229	-0.015773659	-0.053343042
PRRC2A	-0.26067687	0.747100202	0.134000335	-0.21166355	-0.029364624
PARP1	0.568951931	0.68058223	0.444660372	-0.067321142	0.057412495
ATP5I	0.44334403	-0.491965984	0.085763744	0.310437245	0.266780699
TARDBP	-0.077296708	0.690656883	0.060495404	-0.421648117	-0.325668468
CTBP1	0.416890391	0.096161427	0.1428223	-0.060837266	0.038922395
LAMTOR4	0.331701607	0.537130222	0.296680493	-0.087834482	-0.081924464
TMEM123	-0.042148699	0.408174091	0.001511363	-0.315047555	-0.137031931
POLR2A	0.834275162	0.769501705	0.974891299	0.720407515	0.811077806
FNBP4	0.309012509	0.408365137	0.64684179	0.714353034	0.676300258
UBXN1	-0.162908266	-0.476567988	-0.0292926	0.447137264	0.298446884
VPS51	-0.003585282	0.030672941	0.361079422	0.676914462	0.692753486
CSNK2B	0.975655366	0.53296869	0.870886396	0.63189634	0.681777568
HDGF	0.542020362	0.465395441	0.331473781	-0.086642615	0.040303561
SUB1	0.519422542	0.372223826	0.825747814	0.962942408	0.968249261
TYK2	0.166148709	0.376505047	0.038394666	-0.349552184	-0.35072827
C11orf58	0.521590087	0.690949487	0.439332523	-0.058421266	0.011306553
SATB1	0.638471646	0.23344599	0.650449708	0.66908884	0.744053014
MDM4	-0.156734076	0.506518231	-0.176144243	-0.674032291	-0.558160507
EIF3K	0.288184045	-0.452524318	0.275736995	0.739586493	0.61484031
CCNL2	0.217731445	0.383915255	0.019898161	-0.423107205	-0.398268587
SNRNP200	-0.157263174	0.176804776	-0.383874933	-0.79611624	-0.707752412
ZRANB2	-0.03450606	0.685753331	0.029681558	-0.502586727	-0.355758258
SNRPB	0.346348311	-0.167616526	0.266550947	0.446483431	0.485879872
CYFIP2	0.047540969	0.295358496	0.078015237	-0.131185347	-0.183206138
DDX39A	-0.588645085	0.524457171	-0.144755851	-0.36047989	-0.223045754
SH3BGRL3	0.516420734	0.15081682	0.490430416	0.504968033	0.591379959
SRSF7	0.392538296	-0.045224174	0.558612568	0.877001054	0.780576455

RAN	0.572290932	0.140015865	0.370123666	0.246297468	0.337597742
CPSF1	0.092353314	0.388136428	0.107406157	-0.180474973	-0.207314296
GANAB	0.19175526	0.599640136	0.09866581	-0.435585622	-0.295704564
PABPC4	-0.23056202	-0.191492619	-0.130250089	0.055166602	0.132023954
RBM25	-0.407736647	0.31801237	-0.203787019	-0.41414818	-0.417075674
CAPNS1	0.716518238	-0.31409715	0.228097713	0.264498282	0.168312222
UQCRC2	0.324029839	-0.430967408	-0.154124351	-0.135260688	-0.274571416
CLASRP	-0.097540125	0.477920781	-0.15004286	-0.636428215	-0.5542456
FLII	-0.367697162	-0.149095191	-0.633250424	-0.874067712	-0.89281933
SREK1	-0.480508051	0.281093834	0.067483926	0.189391361	0.218138741
RBM17	-0.532414875	0.034884718	-0.647340525	-0.954491758	-0.893131995
UBL5	0.647135168	-0.07975766	0.142172071	-0.05762773	-0.089157147
DUS1L	-0.213394975	-0.223657788	-0.339613539	-0.333887931	-0.243479873
SF3A2	-0.159927295	0.4915574	0.15851263	-0.008639386	0.169662001
SNX2	-0.033737726	0.116781628	-0.341936092	-0.740482116	-0.670828552
SF3B2	0.284036951	0.664892918	0.207125168	-0.338731667	-0.232093497
COX8A	0.870593602	-0.098566291	0.616976408	0.740395055	0.675468056
HCLS1	0.134734779	-0.266965859	-0.373922781	-0.582000184	-0.619240165
DHX15	-0.655597734	0.439354645	-0.169645702	-0.295532917	-0.19379178
ILF2	-0.075548136	0.507184	0.240097777	0.084011394	0.260405616
ATP6V1G1	-0.148076129	0.2985059	-0.139232368	-0.433397229	-0.45051956
XRCC6	0.358543731	0.319879184	0.168278493	-0.165270512	-0.027795597
SLC38A2	-0.854899895	-0.685363862	-0.775697893	-0.392183441	-0.448688708
FXR1	-0.734769569	0.127312616	-0.266166214	-0.169609397	-0.155564656
COX6A1	0.499763271	-0.59242292	0.032651086	0.256883968	0.158040615
P4HB	-0.0046578	0.482349247	-0.109169268	-0.617397901	-0.489879602
AP2M1	0.260202448	0.050528958	-0.0077212	-0.216802543	-0.110986436
ACTN4	-0.035249671	0.311969723	-0.238621367	-0.705364624	-0.625772924
UQCRB	0.556301658	-0.382557677	0.379956188	0.715599804	0.570583833
PARK7	0.874386314	0.027054892	0.688982484	0.771646669	0.752892927
RNF44	-0.148504872	-0.682092811	-0.187736967	0.307807899	0.232986604
SMARCE1	0.299173331	0.709867476	0.621822413	0.419459175	0.588052529
PSMA1	-0.587492666	-0.503005438	-0.339969725	0.131073908	0.033856995
KPNB1	0.54044538	0.670619805	0.405621268	-0.117414013	-0.01496612
PHB2	0.136404971	-0.557239541	0.126459082	0.633410511	0.513944851
STXBP2	-0.759305565	0.032809435	-0.545358939	-0.616022416	-0.627232101
RALY	0.32330528	-0.00914507	-0.059140569	-0.305432925	-0.230167324
FKBP8	0.395483143	0.481433614	0.162120586	-0.338655445	-0.235183275
SLTM	-0.094508007	0.395319053	-0.021516715	-0.320592543	-0.329763907
RBM4	-0.276076741	0.719489014	0.073010075	-0.298729769	-0.196914444
CCAR2	-0.062994042	0.533767494	0.105504428	-0.20721204	-0.016081593
GPX4	0.618628962	0.074607168	0.220365675	-0.017962226	0.037285378
TRA2A	-0.431367222	0.321944246	-0.031552166	-0.068906776	-0.079511938

APRT	0.459531454	-0.051821435	0.547335392	0.821453132	0.821269367
CNDP2	0.2803287	0.739462861	0.346482285	-0.130580267	-0.06338674
SRRM1	-0.836559461	-0.411124025	-0.592431261	-0.280533474	-0.276877511
COX6B1	0.724733793	-0.350538024	0.329677698	0.488024043	0.4123919
COPE	0.007417672	0.618258947	-0.005288563	-0.539028639	-0.401152719
TUFM	0.278402328	0.877743064	0.394309059	-0.152485657	0.005101094
SAFB	0.681158973	0.815523008	0.709946617	0.263222762	0.408396824
H3F3AP4	-0.01737105	0.540481071	0.184044145	-0.088853806	0.100952178
ADD1	0.487624605	-0.201439009	0.000279657	-0.131678123	-0.222120055
PAIP2	0.888029279	0.521513281	0.971959856	0.891794092	0.932039996
ATF6B	0.299066249	0.729883348	0.264594847	-0.291319496	-0.155748178
SPTAN1	0.023321412	0.781239823	0.161616886	-0.36330259	-0.190967806
MARS	0.010162812	0.20044948	-0.066269036	-0.307032377	-0.360377165
STX16	0.071281187	0.386817846	-0.071880272	-0.512813856	-0.482374092
PSMD2	-0.241195673	0.502160316	-0.214981225	-0.693469559	-0.582635312
ENSA	0.957200592	0.414884736	0.91227494	0.823838501	0.852016969
CAP1	0.280758951	-0.174256832	-0.132890196	-0.285250222	-0.38201361
KLC1	0.344414832	0.721405439	0.31267802	-0.219862868	-0.12704427
DBI	0.253877278	0.012214657	0.253664761	0.326522332	0.416534716
TGOLN2	-0.568361286	0.359426141	-0.032140148	-0.014643084	0.086243675
TRIP12	0.281763833	0.86484849	0.420414484	-0.094009258	0.015922018
PSMA7	0.668641166	-0.337996543	0.227568238	0.314645645	0.268655751
NDUFB8	0.672934988	0.194503712	0.321747274	0.043174018	0.113598702
OS9	0.707029844	0.156863393	0.433317747	0.267291187	0.191826677
BCR	-0.50444392	-0.420457397	-0.365812554	-0.038139772	-0.014227982
PPIB	0.228003395	-0.43864864	-0.296717007	-0.343051222	-0.358324475
ARPC4	0.278252254	-0.314608069	-0.007964323	0.076910241	-0.083744557
PRRC2C	-0.509929885	0.447927301	-0.260575737	-0.570290339	-0.505206492
PDCD6IP	0.881193532	0.518236175	0.691224566	0.353795778	0.376482117
CSNK1G2	0.1951411	0.954814832	0.46945524	-0.020508664	0.153196074
MYO9B	0.180821012	0.007377358	-0.213025325	-0.532082374	-0.555999235
PCM1	-0.281903587	0.048570951	-0.28834248	-0.427790369	-0.496046941
OST4	0.925677264	0.043121936	0.571978655	0.498489448	0.448828695
WAC	-0.453967733	0.468750229	-0.267919068	-0.634743266	-0.472590868
DDX42	0.552998815	0.507741067	0.353059028	-0.089890861	-0.060013462
VPS28	0.554946763	0.466296481	0.292178429	-0.173341395	-0.120775956
MAX	0.16948137	0.355219028	0.386369532	0.340230581	0.278395153
ERGIC3	0.30189047	-0.319556359	0.362745692	0.787725095	0.71737851
ATP5L	0.450546835	-0.642579752	0.03821429	0.339526099	0.178828475
SAP18	-0.57184266	-0.404590685	-0.794085169	-0.843004333	-0.91666642
PPP2R1A	0.508157649	0.572148705	0.376940321	-0.068070728	0.078494106
SBF1	-0.089368417	0.516083678	0.110071982	-0.166972519	0.022538886
SF3A1	-0.918245438	-0.534390579	-0.759845759	-0.450199628	-0.472961258

COX7A2	0.854110547	0.079047973	0.744548746	0.847769776	0.833989945
RP11-349A22.5	0.048159591	0.427036955	0.066165826	-0.265868466	-0.274102631
SCAF11	0.367720182	0.476508054	0.265274743	-0.119519422	-0.120517399
DMTF1	-0.022941751	0.42379889	-0.003330286	-0.353665436	-0.351652794
KTN1	0.415258923	-0.179854758	0.126130656	0.137727104	-0.003279706
PNPLA2	-0.592966836	0.449674657	-0.340736547	-0.674555748	-0.551677234
CTNNB1	-0.6718529	-0.229051926	-0.646781527	-0.645026497	-0.719030962
MLF2	-0.621087212	-0.049296184	-0.365387621	-0.283700285	-0.35012354
ACTR3	0.932345283	0.561684582	0.851419405	0.595682676	0.61143831
WDR1	-0.699067156	-0.478505063	-0.794985926	-0.700403956	-0.663290436
CS	-0.20960793	-0.788755395	-0.695407234	-0.549596868	-0.683094167
ARFGAP2	-0.243177936	0.579757165	-0.06119546	-0.461080136	-0.394221538
MTCH1	-0.555534179	0.222253095	-0.392825994	-0.607456236	-0.600329167
AUP1	-0.361849229	-0.283742832	-0.392745156	-0.298849838	-0.431091904
RAD21	-0.07706835	0.355411722	-0.1878084	-0.618908701	-0.581802529
TCERG1	0.441478764	0.7994213	0.511130344	0.040398128	0.217220142
ATP5F1	0.880404906	-0.059836583	0.532821176	0.537637576	0.455664694
ATP6V0B	-0.375812922	-0.228050742	-0.65924146	-0.852603345	-0.892446952
RBM8A	0.323354862	0.716606923	0.793770646	0.73035828	0.812899045
AKAP17A	-0.642186069	0.321701012	-0.422557225	-0.693258852	-0.552767142
DDX6	-0.601400828	0.147234439	-0.124817754	0.004508473	-0.004134923
NDUFA11	0.856881739	0.288818838	0.670055951	0.524395078	0.588448451
CIC	0.281427656	-0.237702342	-0.067705938	-0.101756213	-0.051811699
NDUFA13	0.478551088	0.624267109	0.32263247	-0.200782234	-0.10857796
OXA1L	-0.036376158	-0.628004264	-0.260133192	0.049060204	-0.145713193
UBE2V1	0.393435449	0.742846972	0.36307058	-0.170668182	-0.074449983
YWHAB	0.673106868	0.621208311	0.497319891	0.020554441	0.126644927
COX6C	0.415839539	-0.566325109	-0.034955845	0.156308073	0.095669381
HSPA9	-0.714657548	-0.821398387	-0.721059503	-0.258680306	-0.388655931
MAF1	-0.613455191	0.508015505	-0.174131406	-0.388733666	-0.27996958
U2SURP	-0.17957544	0.833475276	0.236936305	-0.137045317	-0.006832819
PPP1CB	0.910618669	0.259916763	0.876912944	0.915315916	0.902228199
RNF10	-0.666342263	0.159421839	-0.413934704	-0.526419176	-0.528984753
MTPN	0.282226993	0.103873279	0.037022689	-0.193191837	-0.265649689
PPP1R12C	0.261811932	0.414479042	0.018194563	-0.478225145	-0.385153168
UFC1	0.710976467	0.03103656	0.681119107	0.852328169	0.751738487
APH1A	-0.244002908	0.458615955	0.003133353	-0.233838609	-0.228009253
ATP5C1	0.810553506	0.035409635	0.453073519	0.347303682	0.368854674
NCOR2	-0.344984636	-0.535453457	-0.757295411	-0.799047459	-0.808639503
IK	0.60542881	0.000233312	0.180992691	-0.025512458	-0.092202598
GRAMD1A	0.796762211	0.620795954	0.700644304	0.339051688	0.457075802
SH3BGRL	0.401236677	0.096410856	-0.02782965	-0.383674454	-0.338994189
C7orf55-LUC7L2	-0.020953215	0.888727133	0.479574029	0.188583248	0.330430893

DHX9	-0.08096872	0.825343797	0.277880646	-0.110336742	0.081630848
SUPT5H	0.018837269	0.117918942	-0.264119208	-0.624755493	-0.630271954
ANKHD1	0.04629586	0.493382473	-0.03439883	-0.514862159	-0.455683703
HMG20B	0.237568905	0.932252873	0.496123069	0.024464965	0.209996111
CIZ1	-0.025744509	0.403312013	-0.175592431	-0.666658909	-0.577335301
DCTN2	-0.610571065	-0.273442746	-0.438435315	-0.241713973	-0.350464477
HDLBP	0.126925226	-0.241027864	-0.026482941	0.077001958	0.138850434
CPSF3L	0.494008679	0.640848771	0.382747885	-0.106642158	0.039662951
SZRD1	-0.556305969	-0.646590062	-0.642563433	-0.35422958	-0.513918223
FBRS	-0.163635934	0.706379989	0.032698789	-0.435234808	-0.326862923
CSNK1A1	-0.617016164	0.463013373	-0.118967833	-0.240889173	-0.121594197
CAPRIN1	0.289476595	0.62592692	0.259458388	-0.205829891	-0.032834719
NUCB1	0.761883377	0.772477757	0.749153642	0.324297046	0.394284186
CASC3	-0.069195592	0.715839462	0.059307453	-0.448710373	-0.276372641
CTDSP2	0.379325493	0.790208439	0.397071093	-0.136231245	-0.034185931
SRSF4	-0.129373419	0.598904552	-0.005598218	-0.440051836	-0.374259143
PIH1D1	0.456578632	0.557362987	0.326701998	-0.123595349	-0.091924508
SLC25A36	-0.230568394	0.049131722	-0.240210744	-0.366851816	-0.442519355
DDB1	-0.005251768	0.370692079	-0.146474467	-0.593857846	-0.452621092
SORL1	0.391671934	0.071915862	0.00530628	-0.291863334	-0.215868809
BNIP2	-0.916105767	-0.453498352	-0.757640208	-0.517373431	-0.590005636
PDCD4	0.063406025	0.251911811	0.165039369	0.06437747	-0.009402734
PCNP	-0.534407312	-0.251350053	-0.616540236	-0.652737031	-0.739099789
NCOA4	0.018947729	-0.392286219	-0.093692041	0.137063727	-0.039633158
SEC31A	-0.169389185	0.649992666	-0.059495059	-0.561411035	-0.408009233
PSMB1	0.243379636	-0.562686342	-0.151156808	0.031900535	-0.146209222
FIS1	0.994395332	0.437051818	0.883309498	0.725523922	0.745711805
PKN1	-0.016711002	0.520353595	-0.093103837	-0.611290836	-0.488615764
SMARCB1	0.04057831	0.107129654	-0.191294857	-0.484834834	-0.362775988
ALYREF	-0.592026732	-0.170299526	-0.454122135	-0.366967583	-0.272069322
MFSD10	-0.275420062	-0.116761978	-0.584498918	-0.8624558	-0.804070112
NAP1L4	0.377178841	0.419995329	0.111865954	-0.372564465	-0.275721545
SAFB2	0.451493061	0.975153932	0.795186613	0.434702206	0.581551616
IDH3B	-0.805509682	0.040033421	-0.540271126	-0.583491618	-0.585512785
RAB5C	-0.197279057	-0.078791925	-0.508463633	-0.795396017	-0.722683451
ANAPC5	0.836408035	0.226352893	0.531057685	0.318240039	0.27443749
SDR39U1	0.354866254	-0.267342628	0.040954181	0.084276134	-0.066661626

Table S8. Pearson correlation of L- amino acids with IGH representative genes.

Gene	D-Asparagine	D-Glutamine	D-Leucine	D-Phenylalanine	D-Valine
CD74	0.453991425	0.138918741	0.564253851	0.132022631	0.480717593
TPT1	0.213756632	0.689609199	-0.580617003	-0.047615032	-0.565418333

B2M	0.792430115	0.197701485	0.709371545	0.970221819	0.835643844
PTMA	-0.627841895	-0.832504433	-0.091675007	-0.16376044	0.01928238
HNRNPA1	-0.457138968	-0.568067115	-0.816291463	-0.277742647	-0.49036469
HLA-B	0.16334506	0.70221437	0.13635858	-0.307832429	-0.202924408
HNRNPH1	0.228205264	-0.328697922	0.278358931	0.016085666	0.390256421
RPS15A	-0.177870061	-0.097307355	-0.761822168	-0.008220082	-0.492400241
NACA	-0.341910689	-0.25940108	-0.905002763	-0.248051225	-0.621680566
PPIA	-0.469282843	-0.142318769	-0.186401793	-0.070259613	-0.242060222
HLA-A	-0.90442824	-0.314038487	-0.172523163	-0.798061559	-0.467681251
NPM1	-0.553385305	-0.304888931	-0.771492194	-0.286332435	-0.60686985
HMGB1	-0.459782295	0.259976965	-0.479584212	-0.845951139	-0.734548695
HLA-DPB1	0.6627268	-0.15079495	0.015892674	0.6178671	0.436427289
HLA-DPA1	0.873238625	0.385427995	0.514913111	0.573459529	0.591761245
HNRNPA2B1	0.077331212	-0.211230595	0.872077888	0.194080687	0.684909377
MYL6	-0.313530249	-0.69248824	-0.647167881	-0.095858396	-0.247902936
UBB	0.704387291	-0.084922236	0.795074376	0.656476652	0.908244593
PCBP2	-0.479450811	-0.356591757	-0.356971593	-0.728802925	-0.393390948
EEF1D	-0.239748805	-0.362200067	-0.77772769	-0.053383259	-0.426212482
GNAS	-0.010626754	-0.30490364	0.640551614	-0.081431986	0.494761376
RPS13	-0.25701856	-0.118046411	-0.812558562	-0.099150313	-0.561998646
FAU	0.172673393	0.149164086	-0.657250086	0.19992592	-0.358810731
HLA-E	-0.072912274	-0.841374528	0.276550191	0.088559754	0.501198063
OAZ1	0.63999779	0.253661111	0.986736717	0.64267294	0.86142689
SRSF5	0.435175712	0.13689171	-0.272995511	0.074301952	-0.047872595
CFL1	0.090358695	-0.182807052	0.873247684	0.325356925	0.704871395
SF1	0.244099802	0.25322792	-0.366743426	-0.20393668	-0.277542305
FTL	-0.075093538	-0.421565178	-0.60350196	0.167997423	-0.191502804
LENG8	-0.011593138	-0.769359267	0.187613068	0.050874791	0.432697746
EIF4A2	0.313461742	0.558757156	-0.544153827	-0.070619994	-0.458022022
AES	-0.729449774	-0.68769233	-0.720143231	-0.444043657	-0.515940961
HNRNPK	-0.270589846	-0.285214965	0.26980802	-0.463395991	0.089426081
HNRNPC	0.340478925	0.118138245	0.886071161	0.22746023	0.66287
ALDOA	-0.53437842	0.185263936	-0.013993293	-0.34676828	-0.348471813
TPM3	0.505750429	0.342766469	-0.092117777	0.058156054	0.001645654
HNRNPDL	-0.354374787	-0.524076622	0.558454279	-0.027608146	0.418203174
COX4I1	0.573689977	0.532283547	-0.275337172	0.505539242	-0.073578739
PFN1	-0.403276229	0.238417762	-0.178266104	-0.169039986	-0.387026053
HNRNPD	-0.225707015	-0.149461752	0.667019559	-0.180333082	0.349323862
NCL	-0.999417924	-0.45651042	-0.559784728	-0.866501757	-0.694635334
HNRNPU	-0.283876584	-0.121361375	0.567516356	-0.317984965	0.22856762
YBX1	-0.85922014	-0.131010578	-0.251588361	-0.697130449	-0.553671402
NPIPB5	0.361736875	-0.29970271	0.862159779	0.662529247	0.91190557
SERF2	0.110273028	0.01159059	0.668620729	0.456845073	0.534395805

CIRBP	0.541351451	-0.300306666	0.678300167	0.5127198	0.836121613
CYBA	0.151408	-0.084542893	0.837322082	0.442812805	0.688711916
SRSF11	0.641028808	0.381317709	0.122585022	0.211400238	0.197035932
EIF3F	-0.2750068	0.663138596	-0.076055983	-0.553013513	-0.526088788
GMFG	-0.190582142	0.200046335	0.560805733	-0.299897914	0.131789316
NAP1L1	-0.780111337	0.022586581	-0.590181203	-0.645481769	-0.783463621
EIF4G2	0.172686712	-0.259357575	-0.104269268	-0.112629822	0.082674545
EIF3L	-0.255165064	-0.238802413	-0.90722438	-0.300324308	-0.609937176
DDX39B	0.396755576	0.091668129	0.984677568	0.452637333	0.799063422
CCNI	0.39790881	0.727058976	-0.453490336	0.103640558	-0.405506109
EIF4B	0.141781193	0.394720427	-0.74081843	-0.042707231	-0.56891737
YWHAZ	0.725841587	0.143043044	0.278847827	0.422920987	0.462174905
CSDE1	0.791984045	0.349806747	-0.021652643	0.52314784	0.238918702
PFDN5	0.451570478	0.334940294	-0.417307041	0.442227286	-0.137459785
MATR3	0.025492723	-0.227157421	0.243682046	-0.251268517	0.200539886
RBMX	-0.545946025	-0.499836081	0.07294363	-0.628026632	-0.077230913
ATP5G2	-0.569666867	-0.151159341	-0.595669853	-0.266089461	-0.563917289
PSMA6	-0.44449943	0.259272154	0.092050406	-0.691355973	-0.349089325
MORF4L1	0.20792938	-0.509246475	0.386085612	0.133584413	0.545156942
DAZAP2	0.320462485	0.131026967	-0.438865053	-0.031505082	-0.209782341
HSP90AA1	-0.243452299	0.207439025	-0.877908854	-0.541737352	-0.816742455
MYH9	0.54327835	0.73528077	0.614043559	0.20072297	0.315359009
SLC25A3	0.098481266	-0.20427154	-0.690236417	0.05767244	-0.290854955
ATP5B	-0.230862987	0.561953712	-0.400553532	-0.208991411	-0.598738837
PSME1	0.815632147	0.437469409	0.331609736	0.900892905	0.502482669
EWSR1	-0.332572245	-0.531356477	0.501001548	-0.25062229	0.349901219
SET	-0.533585201	-0.210771586	0.405179744	-0.368347801	0.064200327
CALM1	0.298004254	0.658773031	-0.530247145	0.102861959	-0.461436964
CD19	0.056666155	-0.311396156	0.562168526	0.512294442	0.591864972
WIPF1	-0.194512483	-0.939693205	-0.011588604	0.035342676	0.310080382
CD79B	-0.011098399	-0.040192288	0.514716634	0.381898219	0.402103919
RBM5	0.919126063	0.206439807	0.709454799	0.80749264	0.846379342
HNRNPA3	-0.374498904	-0.465183868	0.556809096	-0.209915228	0.349788467
PABPN1	-0.09905435	-0.034258967	0.72489741	-0.119387571	0.396414924
HNRNPM	-0.297018543	-0.470330578	0.437792077	-0.318249184	0.286184038
MAN2C1	-0.213680745	-0.456276628	-0.190611805	0.265621346	0.043133857
SPPL2B	-0.638735982	0.313777714	-0.591716645	-0.662128073	-0.854060588
TMEM259	0.5527245	0.173751857	0.455137709	0.198838771	0.44942266
C19orf43	-0.143171614	-0.370176671	0.073288348	0.358383475	0.213510655
GABARAP	0.491382946	0.1281706	0.950494313	0.666726081	0.840503614
HNRNPUL1	-0.375516096	-0.541679394	-0.017625227	-0.517533626	-0.03112257
TKT	0.254033631	-0.02491692	0.529467501	-0.025499652	0.421443423
CNBP	-0.618440206	0.167472914	0.065037134	-0.65182347	-0.384279913

ARPC2	0.81777081	0.427883139	-0.039711061	0.597762501	0.220439319
COX7C	0.431948828	0.345308147	0.033845208	0.651043897	0.167898521
EDF1	-0.582528924	-0.0878504	-0.064083995	-0.244976011	-0.26566269
ACIN1	0.156071848	-0.319547202	0.880209012	0.40308952	0.804139636
TMA7	0.009489511	0.650863869	-0.126076451	0.065939731	-0.32465513
EIF5A	0.283390801	0.47869846	-0.634844315	0.071138012	-0.463823568
USF2	-0.08161259	0.117404376	0.615744504	-0.243648103	0.249991495
CLIC1	0.122659014	0.034205096	-0.478580844	0.340638466	-0.193542647
HNRNPH3	0.22493635	0.151358845	0.604782111	-0.067610746	0.376835772
U2AF1	-0.813006132	-0.111935159	-0.726479737	-0.981542462	-0.890697094
ARPC3	0.795472143	0.695773597	0.475873286	0.782586977	0.454843021
EIF4H	-0.664128446	-0.296379815	-0.636230501	-0.882398722	-0.686473551
DCAF8	0.400555039	-0.060085064	0.873315937	0.320500604	0.767909449
ARF1	-0.032029435	0.256153487	-0.799776249	-0.351296597	-0.673544041
BAG6	-0.325003508	-0.556245438	0.586053384	-0.042665563	0.45590062
EIF3G	-0.232344715	-0.156658493	-0.696223872	0.011438408	-0.444348767
CHD4	-0.450240652	-0.783717289	-0.207621031	0.035274863	0.033278207
EEF1A1P5	0.343467935	0.159062624	-0.420713104	0.457500686	-0.104761993
CD37	-0.716659381	-0.586744222	-0.152526087	-0.272471187	-0.171754048
RAP1B	0.692167104	0.173541439	0.723472094	0.455875097	0.715378574
CDC37	-0.148604836	-0.096409787	0.671024368	-0.188886065	0.356817191
SMG1P1	0.098607273	-0.802976995	0.32069208	0.460464318	0.649151775
PRKCSH	-0.384082966	-0.163509266	0.555670603	-0.23748895	0.217639979
SRSF6	-0.33407771	-0.714626951	-0.631389824	-0.254238692	-0.268199465
NDUFV1	-0.072904874	0.070598786	0.744871823	0.08491269	0.416105624
PNN	0.25188714	-0.25510665	0.472639583	0.050891998	0.494555363
TMBIM6	0.225058422	0.605010491	0.471071288	-0.149296116	0.094901429
SKP1	0.248378772	0.962240194	-0.076970531	-0.023913782	-0.351269782
CD81	-0.575298894	-0.919378999	0.079144165	-0.183596358	0.174166971
ATP5A1	0.098315578	0.161879021	0.4158069	0.44526795	0.312378183
CHCHD2	-0.419077422	0.106732273	-0.419362894	-0.149469614	-0.48265331
PSMB4	0.570197602	0.810081189	-0.222616868	0.332200824	-0.189801897
ZNF207	0.719594213	-0.113389472	0.591895304	0.609431191	0.792033989
U2AF2	-0.13881222	-0.136087234	0.717564262	0.123625896	0.466842507
TCP1	-0.490717474	-0.075412604	-0.877358167	-0.741300557	-0.831809436
ST13	-0.53750934	-0.019966639	-0.554080444	-0.266666146	-0.580111019
UBAP2L	-0.374587998	-0.086459426	0.401042684	-0.491325934	0.045162419
GDI2	0.321570738	0.770778939	-0.211193478	0.258466756	-0.272678849
MRFAP1	-0.484047604	0.450069448	-0.304380476	-0.47102569	-0.63939407
SNHG1	0.806683197	0.129941622	0.773111348	0.665670797	0.846950867
PAX5	0.777675448	0.512099596	0.754691216	0.50160279	0.637410898
SRP14	0.235390106	0.867115048	0.288302611	-0.1175513	-0.11299576
SASH3	0.503614512	0.950522968	0.261988834	0.108579639	-0.019732551

ARL6IP4	0.045957035	0.433373404	0.528236237	0.193343464	0.201667652
CPNE1	0.612319624	0.531670614	0.788111361	0.682109148	0.624974905
PRRC2A	-0.35087037	-0.672325838	0.303857353	-0.310897412	0.268137554
PARP1	-0.062221443	-0.07236792	0.781424115	0.148956672	0.513067494
ATP5I	-0.523927232	0.163196878	-0.511626418	-0.331382785	-0.636397475
TARDBP	0.411260313	-0.020829685	0.87487617	0.31528689	0.75581859
CTBP1	-0.696529181	-0.042277823	0.020359347	-0.501697115	-0.325296708
LAMTOR4	0.765597666	0.241261718	0.952817241	0.774727336	0.917989425
TMEM123	-0.75993231	-0.386151788	0.095449197	-0.682704891	-0.198971383
POLR2A	-0.071772456	-0.588103441	0.448837398	0.418746837	0.569377896
FNBP4	0.526864084	-0.421732538	0.196893434	0.759561385	0.635148258
UBXN1	0.346370929	0.01772082	-0.542224301	0.310638648	-0.149584602
VPS51	-0.452384572	-0.753192648	-0.657506863	-0.203746713	-0.301876785
CSNK2B	-0.024613809	-0.240672312	0.463564869	0.435239457	0.457882037
HDGF	-0.382868492	-0.062198672	0.49230245	-0.163835338	0.152866159
SUB1	-0.037215136	-0.696304669	-0.137221339	0.387435641	0.262034917
TYK2	0.738409479	0.439873049	0.920665731	0.624209935	0.777936624
C11orf58	0.420572254	0.088459593	0.979938503	0.552482547	0.825990967
SATB1	-0.68257874	-0.564503297	-0.256428285	-0.230321454	-0.223355902
MDM4	0.110871834	0.126600804	0.743921867	-0.048923986	0.43368394
EIF3K	0.003823701	-0.080739039	-0.62422838	0.20033195	-0.307171109
CCNL2	0.563967182	0.452381948	0.924293143	0.469195273	0.68037405
SNRNP200	-0.068082111	0.375601131	0.533977137	-0.277811045	0.093449719
ZRANB2	0.010636417	-0.096285134	0.764897489	-0.03755915	0.502460781
SNRPB	-0.832051668	-0.365202028	-0.599159882	-0.535417928	-0.628754232
CYFIP2	0.943175795	0.332782757	0.746098975	0.817975489	0.828353023
DDX39A	-0.199361298	-0.550433706	0.143783095	-0.324668049	0.173784487
SH3BGRL3	-0.808641577	-0.511058626	-0.32666685	-0.419003889	-0.371551702
SRSF7	0.276710973	-0.287482698	-0.271016243	0.546825997	0.161117143
RAN	-0.730046694	-0.228348985	-0.087107106	-0.408961366	-0.309275997
CPSF1	0.88732491	0.312963001	0.838854721	0.780173146	0.865043757
GANAB	-0.099177196	0.012144303	0.744898489	-0.074366468	0.398659719
PABPC4	-0.957390875	-0.470970946	-0.741391124	-0.876878874	-0.786261371
RBM25	0.723670272	0.14850439	0.586173667	0.458395459	0.653788079
CAPNS1	0.198968557	0.564160477	0.130042054	0.356278894	-0.006089593
UQCRC2	0.625830019	0.886622899	0.300092353	0.510670047	0.153961922
CLASRP	0.335352777	0.251415014	0.839425198	0.161122886	0.565264761
FLII	0.423854603	0.735649126	0.487380736	0.028687507	0.160002131
SREK1	0.079149431	-0.62708939	-0.194199839	0.04745674	0.174479052
RBM17	0.054812899	0.405784045	0.390875296	-0.318037881	0.030952074
UBL5	0.161086833	0.594853357	0.44140703	0.255845884	0.137720335
DUS1L	-0.912954066	-0.110119738	-0.464669981	-0.941604293	-0.760368391
SF3A2	-0.800479554	-0.78151423	-0.148976541	-0.651917685	-0.196436765

SNX2	-0.029472635	0.470829455	0.547804039	-0.210892659	0.090649062
SF3B2	0.225148145	0.089188239	0.925449108	0.256196523	0.662049789
COX8A	-0.014840992	0.047249769	-0.068878832	0.362951647	0.005237651
HCLS1	0.236360168	0.8360101	0.417827904	0.042351103	0.0107337
DHX15	-0.049945849	-0.522933419	0.076002909	-0.207144323	0.199335879
ILF2	-0.813983255	-0.816131209	-0.159588825	-0.618109709	-0.18711588
ATP6V1G1	0.817625031	0.387958331	0.782280089	0.58868256	0.736557383
XRCC6	-0.635976919	-0.127402627	0.228355116	-0.460362595	-0.133371935
SLC38A2	-0.272803202	0.108895323	-0.79315686	-0.618829099	-0.751040547
FXR1	0.182287334	-0.358752956	-0.146210249	-0.03670648	0.115989519
COX6A1	-0.171200528	0.463102949	-0.341068292	-0.046571074	-0.468232118
P4HB	-0.059733875	0.125531079	0.698726855	-0.148337255	0.324254998
AP2M1	-0.725734945	-0.002378412	-0.006876495	-0.608651842	-0.39002366
ACTN4	0.150693166	0.383362781	0.731156256	-0.022446753	0.341502731
UQCRB	0.282139762	0.18460245	-0.276907714	0.500098755	-0.041404579
PARK7	-0.244099743	-0.160195207	-0.11535546	0.194128017	-0.056454653
RNF44	-0.510866025	-0.085750827	-0.96970772	-0.479168956	-0.83944069
SMARCE1	-0.645195727	-0.904537423	0.036964554	-0.275395618	0.098568038
PSMA1	0.051653024	-0.118192376	-0.727944594	-0.121329875	-0.401461025
KPNB1	0.141804606	0.042653774	0.888119826	0.301524861	0.636565156
PHB2	-0.131457237	-0.081322666	-0.769825626	0.009359196	-0.483267915
STXBP2	0.500299988	0.180296451	0.233696337	0.09830491	0.27244522
RALY	-0.506031979	0.244568436	0.155352374	-0.439090254	-0.277069648
FKBP8	-0.018706995	0.185620184	0.759795725	0.049029217	0.391289384
SLTM	0.828202414	0.271024464	0.802764688	0.652342295	0.813027235
RBM4	0.344821627	-0.297167821	0.662075567	0.250451906	0.700893819
CCAR2	-0.746326035	-0.566717455	0.095654126	-0.630528856	-0.11021138
GPX4	-0.377174215	0.207598278	0.244192879	-0.176768599	-0.10859235
TRA2A	0.611541064	-0.191173829	0.28609892	0.448825663	0.565494086
APRT	-0.52928907	-0.539107037	-0.574798219	-0.138758455	-0.3536382
CNDP2	0.560138813	0.013091972	0.984725782	0.604779883	0.921066368
SRRM1	-0.449065787	-0.233305592	-0.766957431	-0.698147487	-0.676488661
COX6B1	-0.149637994	0.268888634	-0.204305457	0.11545062	-0.265891607
COPE	0.007018502	0.005462626	0.769700773	-0.049945086	0.460303843
TUFM	-0.000544008	-0.306325532	0.824465719	0.140736686	0.65531912
SAFB	-0.221507605	-0.422340015	0.631865128	0.143590536	0.504366229
H3F3AP4	-0.790105185	-0.653893268	0.02502452	-0.625597497	-0.133014692
ADD1	0.533619663	0.79239457	0.495848866	0.500922456	0.27595675
PAIP2	-0.094459357	-0.526011862	0.194588065	0.422835033	0.381458563
ATF6B	0.046198219	-0.0600778	0.859429724	0.128676943	0.591445287
SPTAN1	-0.14525578	-0.301573869	0.691091357	-0.107688559	0.469675427
MARS	0.911740098	0.500068079	0.769683169	0.723941205	0.744673009
STX16	0.573492549	0.429331478	0.906613369	0.421395514	0.672963748

PSMD2	0.161096003	0.111009189	0.728680854	-0.030819286	0.452719894
ENSA	-0.112501003	-0.360375279	0.205240065	0.390725944	0.307735652
CAP1	0.7168066	0.837567855	0.583264064	0.573766119	0.392603296
KLC1	0.359932499	0.052962267	0.978182859	0.42309166	0.791452271
DBI	-0.936208423	-0.500289112	-0.505306567	-0.651741103	-0.577872505
TGOLN2	-0.312107317	-0.744632943	-0.238786635	-0.347659673	-0.026813833
TRIP12	0.357183185	-0.188719146	0.945375294	0.459149526	0.87568002
PSMA7	-0.290427641	0.300583135	-0.176127206	-0.060527475	-0.348066222
NDUFB8	-0.367289347	0.115755708	0.314835752	-0.123950371	-0.014093413
OS9	0.705543941	0.444893214	0.658729001	0.849269463	0.648540826
BCR	-0.777537415	-0.341043791	-0.898705945	-0.840756942	-0.861910346
PPIB	-0.332389203	0.584308653	-0.066338816	-0.38084229	-0.475090548
ARPC4	0.866082118	0.71022399	0.314373142	0.776020978	0.374992361
PRRC2C	0.417712936	-0.015807289	0.573087754	0.152116326	0.539600172
PDCD6IP	0.338675283	0.083018887	0.75931932	0.648011087	0.682415146
CSNK1G2	-0.093846451	-0.558959727	0.690935377	0.087826396	0.631668049
MYO9B	0.493060931	0.745774479	0.704888748	0.32110506	0.371983144
PCM1	0.893549787	0.501675686	0.577006465	0.590894009	0.593374136
OST4	0.187823041	0.263215059	0.31366227	0.503325499	0.253220411
WAC	-0.35032788	-0.270457426	0.304141593	-0.502291369	0.067438305
DDX42	0.51079033	0.299819952	0.945502834	0.603139803	0.762290628
VPS28	0.300842921	0.306347721	0.879312557	0.39762689	0.601509675
MAX	0.858002725	-0.038605184	0.492669191	0.904545468	0.810340989
ERGIC3	-0.294530982	-0.329665418	-0.704108822	-0.019584993	-0.407880605
ATP5L	0.242841976	0.576968337	-0.264732809	0.309233258	-0.245496558
SAP18	0.512965468	0.792510905	0.226299998	0.032290768	0.010947918
PPP2R1A	-0.404173886	-0.182409805	0.517743757	-0.170198705	0.20812422
SBF1	-0.77872899	-0.61747456	0.026337011	-0.655821378	-0.148814138
SF3A1	-0.30868803	-0.006940807	-0.711171127	-0.658271329	-0.674944205
COX7A2	-0.250962956	-0.272874026	-0.149457587	0.210751893	-0.030749444
RP11-349A22.5	0.831903778	0.300906816	0.870380843	0.706056921	0.854169689
SCAF11	0.752152134	0.326391344	0.950695462	0.754843723	0.87326168
DMTF1	0.788981053	0.307694154	0.866916387	0.63020641	0.818527306
KTN1	0.856480895	0.654304041	0.433797281	0.832711561	0.478304061
PNPLA2	-0.015648038	-0.174016418	0.394635226	-0.261930584	0.253907608
CTNNB1	0.669116789	0.514714167	0.21698863	0.218310493	0.212177079
MLF2	0.67085905	0.137640237	0.114269894	0.350210876	0.329332864
ACTR3	0.330124862	-0.10887388	0.647148411	0.721480787	0.70326864
WDR1	-0.614130551	0.165975472	-0.519268664	-0.92687402	-0.793258364
CS	0.296348751	0.972288989	-0.10881272	-0.045886524	-0.361544277
ARFGAP2	0.526256873	0.027624146	0.788392334	0.35055935	0.732525119
MTCH1	0.606957518	0.221280335	0.516929479	0.26246989	0.50034571
AUP1	0.891791356	0.577109883	0.230032715	0.560304828	0.344243145

RAD21	0.532818649	0.42041335	0.853776875	0.321557869	0.609632212
TCERG1	-0.388044304	-0.463133898	0.556603098	-0.112199385	0.363221739
ATP5F1	0.295324179	0.319898288	0.245249644	0.580891965	0.243247077
ATP6V0B	0.47834306	0.79143123	0.443226062	0.068122149	0.138202087
RBM8A	-0.065891722	-0.892268473	0.113214608	0.30485029	0.467583918
AKAP17A	-0.34075594	-0.241463941	0.14544865	-0.57152958	-0.051408953
DDX6	0.30758133	-0.369452872	-0.116438322	0.14798386	0.221056956
NDUFA11	-0.439303485	-0.240994557	0.118902217	-0.005082146	0.008274685
CIC	-0.729064852	0.119212309	-0.258052831	-0.615607821	-0.595310723
NDUFA13	0.208826685	0.128883033	0.910967897	0.316513245	0.643058043
OXA1L	0.728514409	0.684621857	-0.11354088	0.538931529	0.026744638
UBE2V1	0.33396836	0.020194425	0.974579747	0.427791107	0.793963353
YWHAB	-0.039760204	-0.022753694	0.754745161	0.206353072	0.496249964
COX6C	-0.403426153	0.370370648	-0.413083377	-0.285580992	-0.60707898
HSPA9	0.081681885	0.341108034	-0.723592593	-0.269548029	-0.603623792
MAF1	0.038077884	-0.43151739	0.249177921	-0.138023207	0.313914407
U2SURP	0.177728447	-0.512441348	0.602008776	0.187833435	0.677928441
PPP1CB	-0.031927525	-0.321383818	0.054587085	0.451784051	0.24159565
RNF10	0.559253322	0.107576503	0.334591382	0.209855572	0.407282128
MTPN	0.864138319	0.635302839	0.766745659	0.761127306	0.677883595
PPP1R12C	0.052315917	0.285261068	0.768043716	0.033833013	0.378912408
UFC1	0.424894616	-0.048497984	0.049300747	0.747910479	0.353815903
APH1A	0.750987499	0.027163168	0.660338263	0.584564323	0.783339496
ATP5C1	-0.347894274	0.083967878	0.094775672	-0.018488415	-0.107508924
NCOR2	-0.257429524	0.668564979	-0.11474505	-0.577996526	-0.550410759
IK	0.562712355	0.657970988	0.63364775	0.607156305	0.446364835
GRAMD1A	-0.317110214	-0.312057245	0.478891057	0.08170374	0.318885648
SH3BGRL	-0.051775184	0.478411342	0.533768706	-0.041733693	0.104337116
C7orf55-LUC7L2	-0.021177554	-0.789599085	0.413708067	0.147096607	0.594476376
DHX9	-0.380456641	-0.701578293	0.375182092	-0.25867206	0.323046447
SUPT5H	0.551769528	0.661103507	0.771592411	0.325435249	0.469468139
ANKHD1	0.48455428	0.302535487	0.919621727	0.354098226	0.686425266
HMG20B	-0.253441549	-0.619214013	0.595952606	-0.029605195	0.516344433
CIZ1	0.179252637	0.307681456	0.781608981	0.026472614	0.423193437
DCTN2	0.684247463	0.27270662	-0.044575312	0.344596397	0.181334023
HDLBP	-0.925649318	-0.205532089	-0.564534255	-0.770878759	-0.75342384
CPSF3L	-0.280902553	-0.156220154	0.637186261	-0.069388363	0.335591482
SZRD1	0.635531082	0.635903258	-0.195862373	0.235303781	-0.097989818
FBRS	0.338471308	-0.108774471	0.809808637	0.232659773	0.708734324
CSNK1A1	-0.196349933	-0.619850361	0.007702652	-0.305429778	0.123816145
CAPRIN1	-0.470611512	-0.282606762	0.487685091	-0.304440374	0.179487998
NUCB1	0.323781363	-0.15493919	0.864600284	0.635347396	0.835315996
CASC3	-0.191429785	-0.268949794	0.632467838	-0.198887311	0.385639995

CTDSP2	0.335109161	-0.048770664	0.973412518	0.441043952	0.82429372
SRSF4	0.547863523	0.081628196	0.862626635	0.402499509	0.774130608
PIH1D1	0.589128758	0.262187189	0.980510721	0.646036329	0.836172282
SLC25A36	0.923373461	0.499453435	0.57951108	0.643783386	0.617223316
DDB1	-0.35210736	0.059192318	0.474641564	-0.405946126	0.056118302
SORL1	-0.404091108	0.265939998	0.285099505	-0.322603141	-0.146074339
BNIP2	0.271580193	0.23930666	-0.341315701	-0.171202	-0.239803386
PDCD4	0.961159999	0.226115444	0.602233218	0.882192842	0.80315905
PCNP	0.7415705	0.657520564	0.322103964	0.305616765	0.262238541
NCOA4	0.86767139	0.546909795	0.073340995	0.724590094	0.284557215
SEC31A	-0.061314285	-0.144145078	0.67016158	-0.149167806	0.415558051
PSMB1	0.679918039	0.815813398	0.080152537	0.564578156	0.078973531
FIS1	0.039291831	-0.208625159	0.379278856	0.505308509	0.430744609
PKN1	0.034172056	0.126480027	0.755562827	-0.064464499	0.409626041
SMARCB1	-0.631259545	0.066782971	0.128711959	-0.640090595	-0.306910531
ALYREF	-0.842340079	-0.339478926	-0.595891173	-0.967761092	-0.726633258
MFSD10	-0.198819373	0.498665878	0.262783406	-0.472529396	-0.20858867
NAP1L4	-0.032563455	0.237227939	0.7268086	0.015945674	0.338564142
SAFB2	-0.086898653	-0.762439667	0.544910958	0.273182176	0.664412898
IDH3B	0.416473695	0.083360326	0.149894636	0.026215697	0.21561481
RAB5C	-0.304858904	0.425187988	0.237012142	-0.522457977	-0.243277074
ANAPC5	0.503741033	0.355036282	0.649147926	0.729451095	0.581411572
SDR39U1	0.844956714	0.712846559	0.374178417	0.783541138	0.404175116
